# Resident travel mode prediction model in Beijing metropolitan area

**DOI:** 10.1371/journal.pone.0259793

**Published:** 2021-11-11

**Authors:** Xueyu Mi, Shengyou Wang, Chunfu Shao, Peng Zhang, Mingming Chen

**Affiliations:** 1 Key Laboratory of Transport Industry of Big Data Application Technologies for Comprehensive Transport, Ministry of Transport, School of Traffic and Transportation, Beijing Jiaotong University, Beijing, China; 2 School of Civil Engineering, North China University of Technology, Tangshan, Hebei province, China; 3 Engineering General Contracting Department II, Beijing Municipal Road & Bridge Co., Ltd., Beijing, China; Central South University, CHINA

## Abstract

With the development of economic integration, Beijing has become more closely connected with surrounding areas, which gradually formed the Beijing metropolitan area (BMA). The authors define the scope of BMA from two dimensions of space and time. BMA is determined to be the built-up area of Beijing and its surrounding 10 districts. Designed questionnaire survey the personal characteristics, family characteristics, and travel characteristics of residents from 10 districts in the surrounding BMA. The statistical analysis of questionnaires shows that the supply of public transportation is insufficient and cannot meet traffic demand. Further, the travel mode prediction model of Softmax regression machine learning algorithm for BMA (SRBM) is established. To further verify the prediction performance of the proposed model, the Multinomial Logit Model (MNL) and Support Vector Machine (SVM), model are introduced to compare the prediction accuracy. The results show that the constructed SRBM model exhibits high prediction accuracy, with an average accuracy of 88.35%, which is 2.83% and 18.11% higher than the SVM and MNL models, respectively. This article provides new ideas for the prediction of travel modes in the Beijing metropolitan area.

## 1. Introduction

With the development of society, between urban and cities, the links between urban and regions are increasingly close. The central status and scope of the city are more prominent in the region. An advanced city form—Metropolitan area, gradually formed. The urban circle can maximize resource allocation of regional economic development. At the same time, traffic problems in the Metropolitan area have also become more intensified. Existing transportation services are difficult to fit the contact and development of the Metropolitan area, leading to severe traffic congestion, greatly affecting the various functional functions in the Metropolitan area. Residents in the Metropolitan area, as an important assumption in urban construction, and urban function operation, are facing more severe traffic issues. They usually close up with the construction, service and operation of the city. However, there is currently a lack of scientific system research in daily activities in the Metropolitan area. For the city of Beijing, With the evacuation of "non-capital functions" in Beijing, the problem of lack of transportation channels between Beijing and surrounding cities has gradually been exposed, which has led to the problem of unsatisfied travel needs and traffic congestion. It is necessary to study the travel characteristics of residents in surrounding districts to commute to Beijing. The paper will provide some suggestions to solve the dilemma that inconvenient travel and long commuting time. These suggestions can help to accelerate the construction of smart transportation cities in Beijing Metropolitan area. The study for residents’ mode choice in the Beijing Metropolitan area will help to promote the construction of a harmonious society. It is also important for enhancing traffic demand forecast analysis and demand management measures.

To study the travel characteristics of residents, the Beijing metropolitan area is selected as the research scope. However, there is no consensus on the definition of the metropolitan area. It is chaotic for the scope of the Beijing metropolitan area. Therefore it is necessary to define the scope of the Beijing metropolitan area. Japan first introduced the metropolitan area and defined the metropolitan area by dividing the travel range of residents in a day as the boundary [1]. Zhao et al. (2018) considered economic and historical factors to define Beijing and Tianjin as the core capital area and analyzed the development trend of the future metropolitan area. Pan et al. (2018) proposed the concept and classification system of metropolitan areas [2]. Zhejiang Province was taken as an example. Three types of metropolitan areas, quasi-metropolitan areas, and potential metropolitan areas are divided. Xu et al. (2019) constructed a system of general ideas and technical methods for the delimitation of the metropolitan area [3]. Xuzhou was taken as an example to verify the scope from quantitative and qualitative methods.

From the perspectives of commuting and consumption, Wang et al. (2018) used mobile phone signaling data to define the scope of the Shanghai metropolitan area and compare it with foreign metropolitan areas [4]. Li et al. (2010) used the data envelopment analysis method combined with fuzzy mathematics to study the relationship between the spatial distribution of residence and employment and commuting [5]. Although scholars as mentioned above, have considered the scope of the metropolitan area from different perspectives, too many perspectives have problems with low adaptability. For the scope of the Beijing metropolitan area, this article is trying to define it in representative and straightforward aspects. The definition of the Beijing metropolitan area is conducive to study the residents’ travel behavior, which in turn facilitates the development of target traffic planning, traffic policies and urban layouts. It is conducive to alleviating the pressure on the surrounding traffic, and promoting economic development.

At present, China is actively integrated into the global production network, especially in large cities. Due to the diversification of urban functions, urban population continuous growth and construction space spread rapidly, enabling the function and supporting facilities of the domestic central cities to overload. The environment is constantly deteriorating, and a series of urban issues are concentrated. Therefore, research metropolitan area traffic travel issues help relieve traffic congestion and accelerate urban development processes. Scholars have more research on the city’s evolution [6, 7] However, Lambregts (2009) correctly indicates that multi-layer and interdependence between the cities that can see in a particular area relies on the metrics used to measure its indicators [8]. De et al. (2010) thought urban networks are multiplexed phenomena, so multi-level and interdependence can be studied by assessing different types of functionality between urban and regions [9].

For the factors affecting mode choice of residents, personal attributes [10, 11], family attributes [12, 13], and travel attributes [14] in the previous literature are found to have influence. Zhang et al. (2017) selected three spots for the questionnaire survey in Beijing, and factors including age, car ownerships, and monthly income have a significant impact on the choice of the travel mode [15]. For commuting mode choice, Ding et al. (2021) design the survey to capture the data of households (e.g., household size, income, ownership of vehicles), individuals (e.g., occupation, gender, age, driving license, and education), and travel diaries (e.g., departure time, travel origin, purpose, arrival time, travel mode, and destination) [16].

In the early analysis of travel behavior characteristics, aggregate data is mainly used. The characteristics of travel behavior can be predicted by fitting several parameters to a simple model. However, with the increase in the types of travel modes and the changes in external objective conditions, the factors affecting travel behavior have become more and more complex. Traditional travel analysis approaches cannot explain the mechanism of travel characteristics well. Therefore, scholars have begun to explore new theoretical methods to analyze the characteristics of travel behavior through considering more complex factors.

Subsequently, travel surveys and special surveys on travel behaviors carried out in many cities gradually developed. Based on the survey data, the dis-aggregated model theory was used to study the travel behavior and obtained rich research results. Scholars [17–21] have used dis-aggregate models to study the characteristics of individual travel patterns. A theoretical system of dis-aggregated models was constructed. The theoretical system included the Multinomial Logit Model (MNL) and Nested Logit Model (NL) [22–29].

In addition to the above statistical models, machine learning models are also widely used to analyze travel characteristics of residents. Machine learning models included Support Vector Machine (SVM), the K-nearest neighbor, the random forest and Softmax regression [30–33]. Although SVM has unique advantages in dealing with small sample, nonlinear and high-dimensional pattern recognition problems, its classification accuracy is not high for more complex classification problems, and the processing cost of current big data classification is too large [34]. The algorithm process of K-nearest neighbor is simple and easy to understand. However, K-nearest neighbor is a lazy learning method. When the data is unevenly distributed, the classification error rate will increase, and the calculation complexity of the classification process changes large. Random forest has the advantages of strong computing power, high accuracy, fast training speed. However, the random forest will produce some relatively large noise classification problems, limiting its application in the prediction of travel mode. These shortcomings cause the above three classifier models to have certain limitations in application. The Softmax regression classifier has the advantages of many types of classification, simple application, high accuracy, good training [35, 36]. At present, few scholars apply Softmax regression classifier to predict the travel mode of residents. It is meaningful to introduce the Softmax regression to predict the travel mode in Beijing metropolitan area.

The rest of the paper is organized as follows: Section 2 is the definition of Beijing Metropolitan Area. Section 3 analyzes the travel characteristics in metropolitan areas. In Section 4, methodologies provide details on travel mode prediction based on Softmax regression in Beijing metropolitan area. Section 5 presents the modeling results. To verify the prediction accuracy, the prediction results were compared with the SVM and MNL models. Finally, Section 6 summarizes the paper and highlights the perspectives from the viewpoints of the research.

## 2. Definition of Beijing metropolitan area

At present, the definition of the metropolitan area has not formed final conclusions. Due to its remarkable similarity with urban agglomeration and its different technical concepts, many studies lack the prerequisite explanation of the spatial scale and confuse the two concepts. The following will summarize the characteristics of metropolitan areas and urban agglomerations, distinguish metropolitan areas and urban agglomerations, and provide a theoretical basis for the definition of Beijing metropolitan areas.

The metropolitan area is generally formed by the core city and the adjacent small and medium-sized towns with close social and economic ties with this core city and tend to be co-urbanization [1]. Affected by the dual constraints of commuter travel time (according to 1h) and travel cost, the spatial boundary of the metropolitan area should be around 70km. The population and transportation demand characteristics of metropolitan areas and urban agglomerations are shown in Table 1.

**Table 1:** Comparison of population and traffic demand characteristics between metropolitan areas and urban agglomerations.

Main characteristics	Population size	Transportation demand characteristics
Metropolitan area	total population ≥ 5 million	commuting transportation needs
	Central city population	
	≥ 1 million	
Urban agglomeration	total population ≥ 25 million	the transportation of raw materials and semi-finished products between the upstream and downstream industrial chains; A large number of business trips between cities

The initial stage of urban agglomeration is the discrete development of individual cities, and the advanced stage is the development of urban clusters. The growth experience of urban agglomerations is: "towns—central cities—metropolitan areas—urban agglomerations." Combined with related research, urban agglomerations have a broader spatial scope than metropolitan areas. Urban agglomerations are tightly integrated areas composed of at least two metropolitan areas and their surrounding small and medium cities. The spatial structure of urban agglomerations is multi-centered. The network structure has a significant "circle + corridor" effect, which presenting a development law that extends from the distribution of the metropolitan circle to the axis [37, 38]. The spatial structure of a more mature urban agglomeration mainly includes three aspects: the central city metropolitan area, the main axis of the urban agglomeration, and the medium and small cities and town networks. The scope and structural characteristics of metropolitan areas and urban agglomerations are shown in Table 2.

**Table 2:** Comparison of the scope and structural characteristics of metropolitan areas and urban agglomerations.

Main characteristics	Scope scale	spatial structure characteristics
Metropolitan area	Combined commuting time and commuting expenses, Generally around 70km	layered structure
Urban agglomeration	Huge space	circle layer + corridor structure

**Fig 1:**
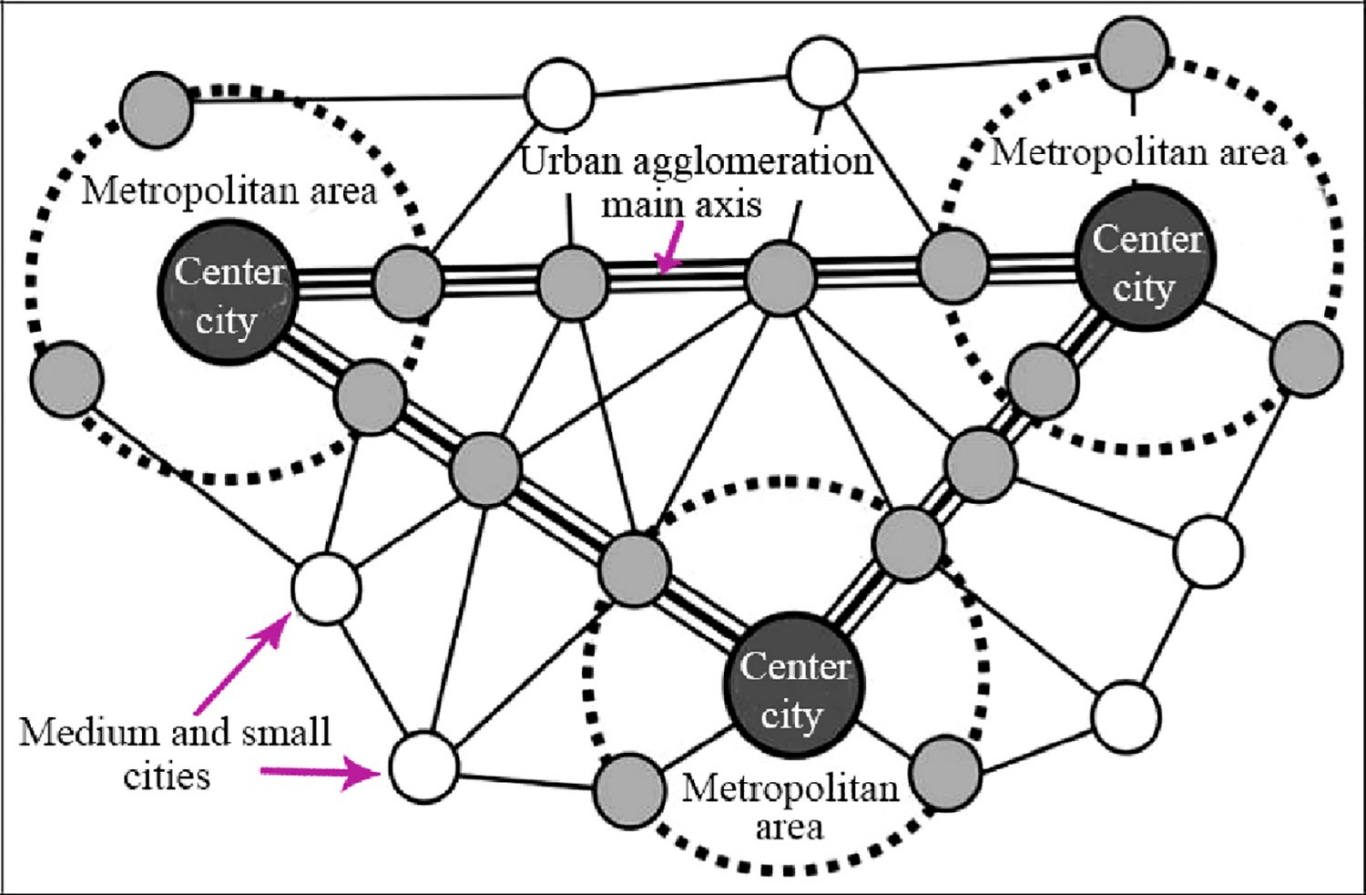
Urban agglomeration and metropolitan coordinating region structure.

The structural characteristics of metropolitan areas and urban agglomerations are shown in Fig 1.

There are many methods for defining metropolitan areas. The definition of dominant elements is complicated. The metropolitan area is the production of the interaction between cities. The factors that affect the interaction between cities are diversified. To avoid the above mentioned complicated factors, the article reference the definition of the metropolitan area introduced by Sun et al. (2003) [10]. Combined with the situation in Beijing, the scope of the Beijing metropolitan area is considered comprehensively in terms of space and time.

### 2.1. Space element

When considering the definition of spatial elements, it is necessary to comprehensively consider factors such as the accessibility, convenience, travel cost, and transfer times of the residents in the metropolitan area to determine a metropolitan area radius that meets actual experience, and divide a rough the circled metropolitan area. Then, it is necessary to check whether there are natural obstacles that are not conducive to traffic within the scope. If it exists, the radius should be reduced on the side of the natural obstacle and multiplied by an appropriate weight coefficient. Finally, the accurate metropolitan area can be obined by taking the administrative boundary of each city as the boundary of the metropolitan area. References, and conclusions determine that the radius of the metropolitan area centered on Beijing is about 70km and the total area is about 20,000km^2^.

### 2.2. Time element

The prominent role of the time element is reflected in the delineation of the "one-hour metropolitan area" and the "one-day exchange circle." The connotation of the time element is that the one-way travel time required for residents in the central city and surrounding towns within the metropolitan area is one hour. When defining the scope of a metropolitan area, it is necessary to understand the main modes of transportation for residents of the central city and surrounding districts and counties. Secondly, it is needed to understand the traffic network and road speed corresponding to each mode of transportation. Finally, according to the transportation network of different levels, the maximum distance reached within one hour from the central city is obined. At the same time, based on the administrative boundary, the scope of the metropolitan area can be determined. When defining the scope of the Beijing metropolitan area based on time elements, it is essential to consider the road network corresponding to several modes of transportation: buses, subways, cars, buses, and railways. Refer to the discussion of Chen et al. (2016) on the scope of "1 hour metropolitan area" in Beijing [39], the definition of spatial elements embodies the influence of distance and region. The definition of time elements reflects the convenience of external transportation links in the central city.

The scope of the metropolitan area selected by combining the two factors. The Beijing metropolitan area mainly includes the main urban areas of Beijing, Wuqing, Sanhe, Main urban area of Langfang (hereinafter referred to as Langfang), Zhuozhou, Gu’an, Dachang, Yongqing, Yanjiao, Huailai, and Xianghe. The space element mainly considers the geographical factor of distance. The time element mainly considers the convenience of travel. The scope of Beijing metropolitan area belongs to the core area of government planning and development. In the following, a questionnaire survey will be conducted on the Beijing metropolitan area to study the travel characteristics of residents.

### 2.3 The description of the studied area

The studied area can be divided into four medium regions, namely Beijing, the northern region, the southern region, and other regions. The social and economic development and transportation systems of the studied area are summarized.

#### 2.3.1 Beijing

By 2020, the total production value of Beijing has increased by 6.1% to 3.18 trillion RMB. According to the National Bureau of Statistics, Beijing’s social and economic development index is shown in Table 3.

**Table 3:** Beijing’s social and economic development index.

Index	2015	2016	2017	2018	2019	2020
Gross product (billions of RMB)	2,296.9	2,457.9	2,638.4	2,820.8	2,998.7	3,177.9
GDP growth rate in the actual area	6.9%	6.5%	6.3%	5.6%	5.4%	4.7%
Personal consumption (billions of RMB)	849.5	934.8	1,024.9	1,124.2	1,227.2	1,333.7
Population (million)	22.8	22.4	22.8	23.1	23.4	23.6
Gross the per capita area (RMB)	100,639	109,693	115,956	122,273	128,377	134,539
Actual pay (year-on-year)	7.5%	7.1%	6.3%	5%	4.5%	4.5%

At the end of 2020, there were about 20 million people in Beijing, and 1,345.25 million household registration population. There are eight national expressways in Beijing and the external area, namely Jingha (G1), Jingtai (G3), Jinggangao (G4 Jingshi), Jingkun (G5), and Daguang (G45 Beijing, Jingcheng) Highway, etc. Beijing has been linked to ten districts through the peripheral expressways.

#### 2.3.2 The northern region

The northern region includes Sanhe, Yanjiao, Xianghe, and Dachang. The northern region is close to Beijing, just 40 kilometers away from the center of Beijing, approximately 50 kilometers away from the capital international airport. The districts of the northern region are in the gold node of the "one hour economic circle." As of the end of 2020, Sanhe (including Yanjiao) household registration population is about 650,000. The total production value is about 60 billion RMB. The total production value of large factory counts reached 8.8 billion RMB. The total production value of Xianghe was about 20 billion RMB. The overall economic form of the northern region has been well operating and has a rapid growth situation.

There are three kinds of mode choices for residents in the northern region, namely subway, bus, and passenger car. The bus operating in the northern region includes No. 930, NO. 938 and other lines. The endpoint station is near the economical center of Beijing. Passenger car enter and leave Beijing mainly though Tongyan Expressway, Beijing Ping Expressway, Jingqin Expressway, etc.

#### 2.3.3 The southern region

The southern region includes Langfang, Gu’an, and Yongqing. The southern region is located in the southern area of Beijing, about 50 kilometers away from Beijing’s center area. It is known as the "Golden Zone." As of the end of 2020, The GDP of Langfang is about 32.4 billion RMB. Gu’an is a "zero distance" district in the capital, with a regional GDP of about 20 billion RMB. Yongqing is strategically located, with a regional output value of about 10 billion RMB. The geographical advantages of the southern region promote rapid economic and social development.

There are many bus lines for residents of the southern region to enter Beijing. In addition, there are two train stations, named Langfang Station and Langfang North Station in the Langfang, with a total of 30 trips entering Beijing throughout the day. Residents enter Beijing through Jingtai Expressway, Jinghu Expressway, and Jingkai Expressway.

#### 2.3.4 Other regions

Other regions include Zhuozhou, Wuqing, and Huailai. Zhuozhou is adjacent to Gu’an and Langfang to the east, and is only 62 kilometers away from Beijing’s Tiananmen Square. The total population of the city is more than 570,000 people, and GDP is 30.1 billion RMB. Wuqing is 71 kilometers away from Beijing, with a registered population of 925,000, and its annual GDP is 114.499 billion RMB. The eastern part of Huailai is bordered by the Yanqing, Changping and Mentougou districts of Beijing, with a total registered population of 360,000 and a GDP of 14.5 billion RMB. Huailai is the “first gateway” to the west of Beijing.

There are three expressways running from north to south in Zhuozhou, and two railway transport stations. There are six expressways in Wuqing District. The train line of Jingjin special line stops 23 times per day in Wuqingri, and it takes only 23 minutes from Wuqing to Beijing. Huailai is located in the golden zone of Beijing’s one-hour economic circle, only 60 minutes drive from Zhongguancun, which is an economical center of Beijing. The surrounding traffic conditions are relatively convenient. The Jingzhang Expressway and the Jingzhang High-speed Railway are connected with each other. The transportation network is densely woven, making it convenient for residents to enter Beijing.

## 3. Travel characteristics in Beijing metropolitan areas

### 3.1. Questionnaire

#### 3.1.1 Survey scope

The scope of the investigation is defined as the surrounding 10 districts and counties in the Beijing metropolitan area, namely: Sanhe City, Langfang City, Dachang County, Xianghe City, Zhuozhou County, Gu’an County, Yongqing County, Huailai County, Yanjiao County, Wuqing County.

#### 3.1.2 Survey content

The survey is an individual travel survey of residents. It is divided into two types: manual questionnaire survey and online questionnaire survey. The manual questionnaire survey is processed on the street. We conducted this survey in 2018. The survey content is mainly divided into three parts: personal attributes, family attributes and travel attributes. Personal attributes include residential address, individual age, and commuting frequency. Family attributes include car ownerships, and disposable income. Travel attributes include travel time, and travel purpose.

#### 3.1.3 Investigation method

To ensure the validity of the questionnaire, the resident travel survey adopts a simple random sampling survey method. The manual questionnaire survey adopts an on-site survey. The survey site randomly selects the surveyed persons and asks questions about the survey items. The investigator fills out the questionnaire. In addition, the online questionnaire survey adopts the principle of random distribution on the Internet to survey the residents who live in the Beijing metropolitan area.

#### 3.1.4 Number of questionnaires

**Fig 2:**
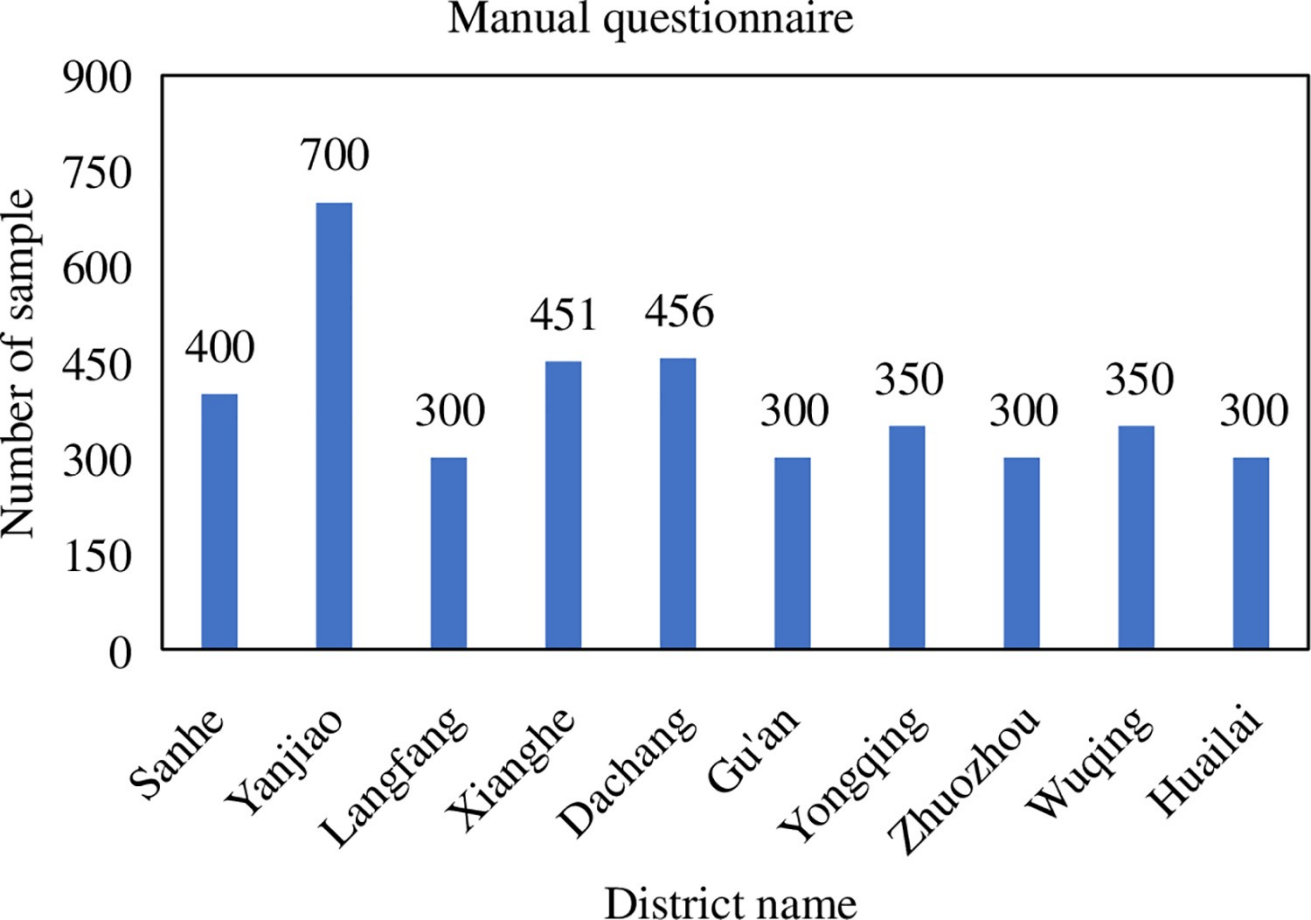
Survey sample size—artificial questionnaire.

**Fig 3:**
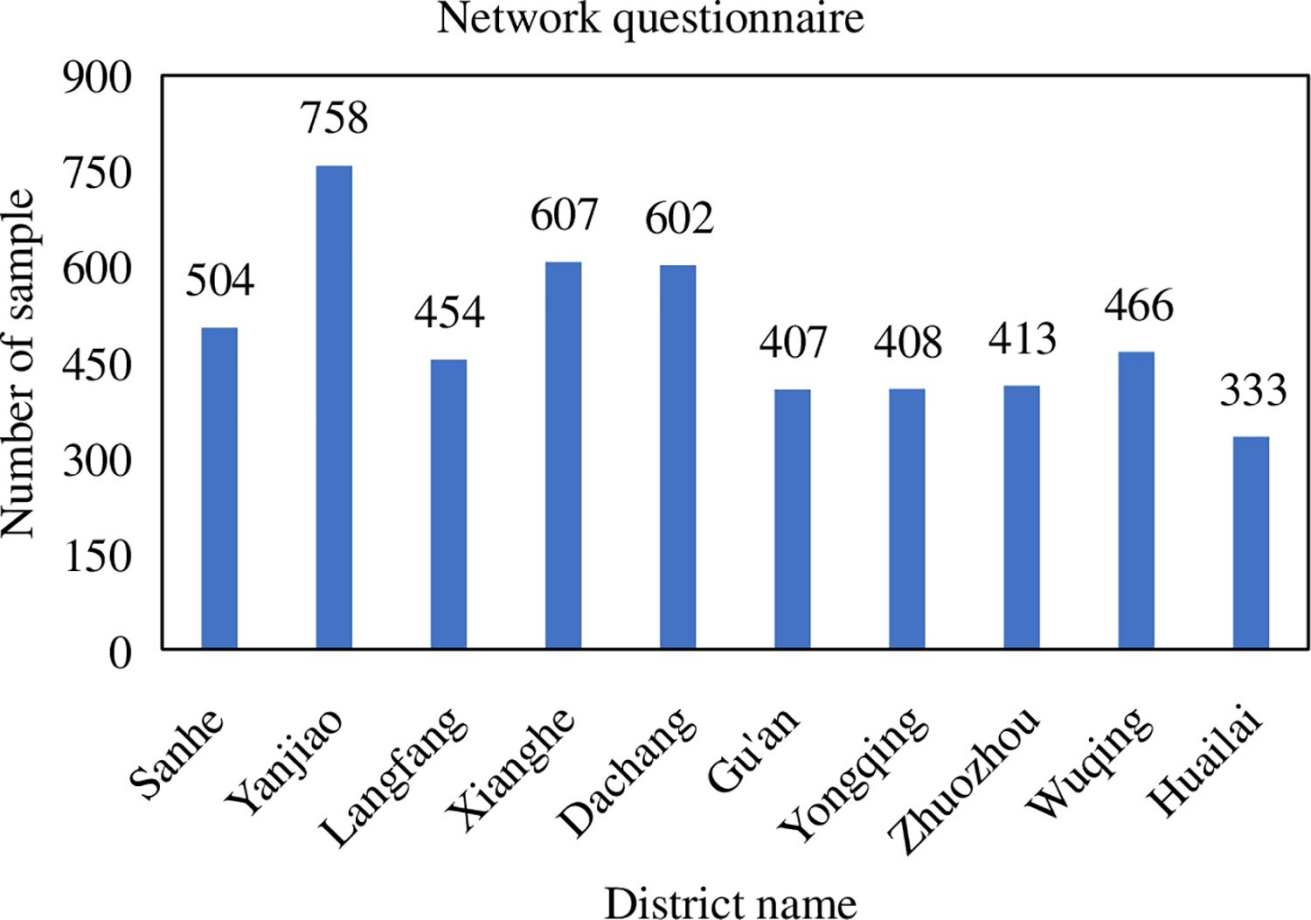
Survey sample size—online questionnaire.

The number of questionnaires is determined by the population of the studied districts. The proportion of the sample is 0.5% of the population. A total of 3907 valid questionnaires were collected from the manual questionnaire surveys conducted by residents of various districts and counties in Beijing, specifically: 400 in Sanhe City, 700 in Yanjiao County, 300 in the main urban area of Langfang City, 451 in Xianghe County, 456 in Dachang County, and Gu’an County 300 samples, 350 samples from Yongqing County, 300 samples from Zhuozhou City, 350 samples from Wuqing District, and 300 samples from Huailai County. The specific number of questionnaires is shown in Fig 2. The online version recovered 4,952 valid questionnaires. The specific number of questionnaires is shown in Fig 3.

### 3.2. Travel characteristics analyze

**Fig 4:**
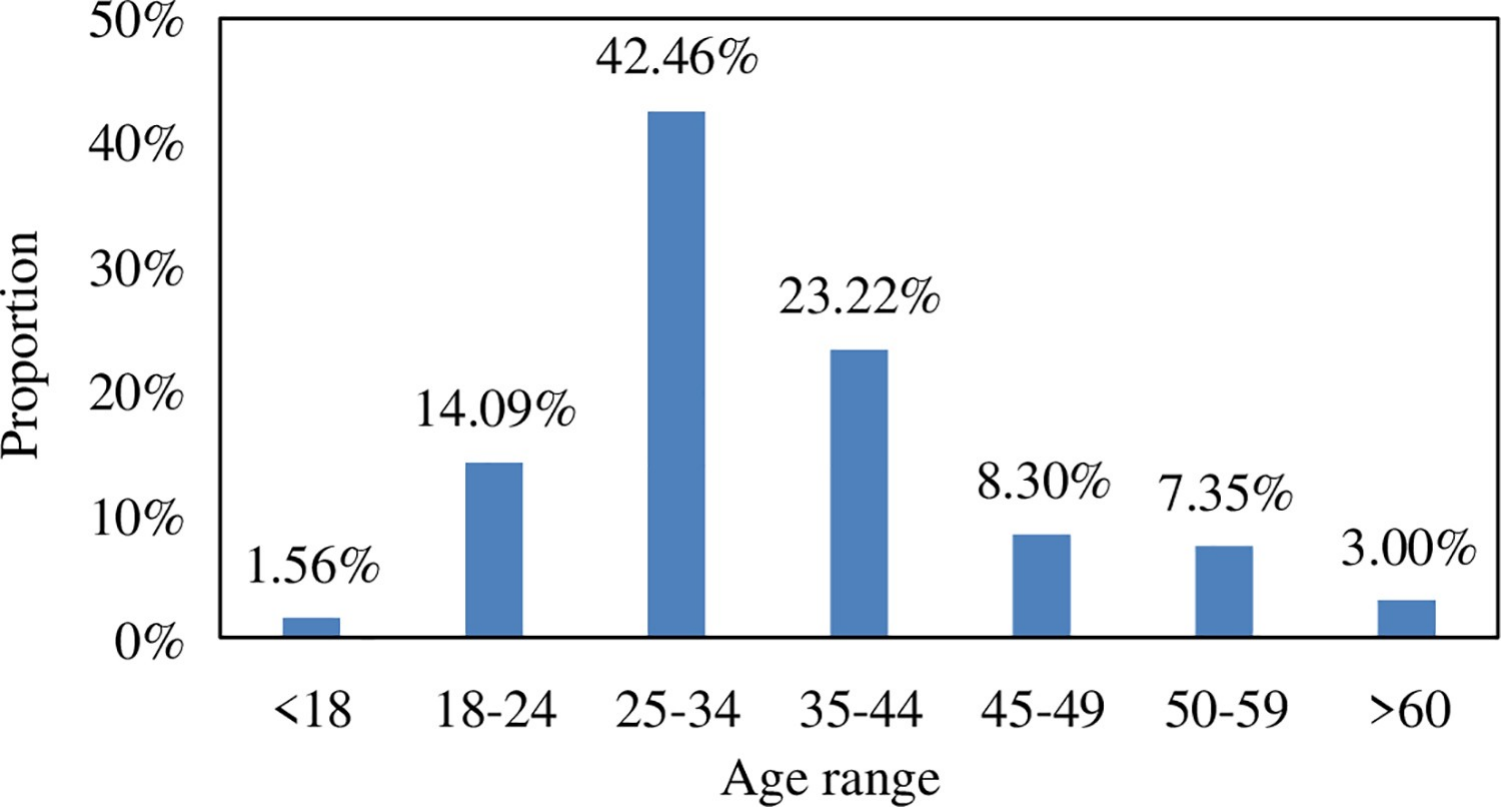
Beijing metropolitan population age distribution.

The survey data collected from the questionnaire was used to obtain the basic information about the age of residents in the Beijing metropolitan area, disposable income per month, number of vehicles, travel time, and weekly travel frequency. The age distribution of the surveyed residents is calculated, and the age distribution of residents in the surrounding districts and counties in the Beijing metropolitan area is shown in Fig 4.

According to statistics on the age distribution of the surveyed persons, residents aged 25–34 accounted for the highest proportion of all surveyed persons, accounting for 42.46%. The age range in 35–44, 18–24, 45–49, 50–59, 60 and above, and under 18, accounting for 23.22%, 14.09%, 8.30%, and 7.35% 3% and 1.56%. The survey samples have a strong age dispersion and have certain pertinence.

**Fig 5:**
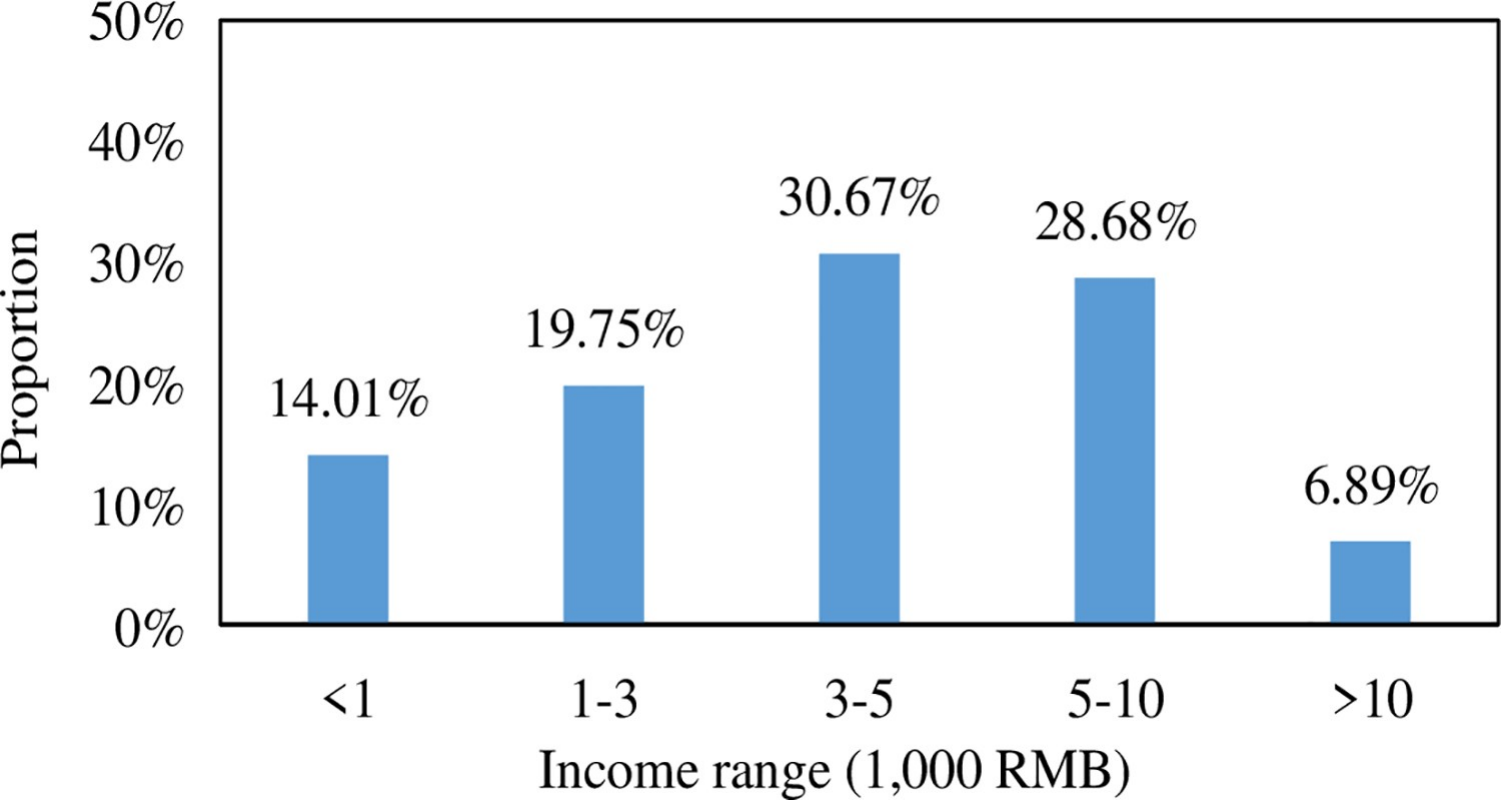
Beijing metropolitan population disposable income distribution.

The distribution of disposable income of residents commuting Beijing is calculated, and the distribution of disposable income is shown in Fig 5.

According to the statistics of the surveyed persons’ disposable income distribution, it is found that the disposable income of all surveyed persons presents a normal distribution of "small at both ends and big in the middle." Disposable income of 3,000–5,000 RMB accounted for the highest proportion, accounting for 30.67%, followed by disposable income of 5,000–10,000 RMB, 1,000–3,000 RMB, less than 1,000 RMB and more than 10,000 RMB, accounting for 28.68% and 19.75%, 14.01%, and 6.89%, respectively.

The vehicle ownership of residents entering Beijing is calculated, and the vehicle ownership of residents in the surrounding districts in the Beijing metropolitan area is shown in Table 4.

**Table 4:** Population vehicle ownership distribution and travel time distribution in beijing metropolitan area.

Beijing metropolitan passenger car ownership distribution									
**Vehicle presence**			**No vehicle**				**Own vehicle**		
Proportion			37.04%				62.96%		
Beijing metropolitan resident travel time distribution									
**Period**	**<30 min**	**30–45 min**		**45–60 min**	**60–90 min**	**90–120 min**		**120-180min**	**>180min**
Proportion	1.17%	1.03%		13.79%	14.35%	32.86%		27.95%	8.85%

According to the vehicle ownership statistics of the surveyed persons, 62.96% of residents in the Beijing metropolitan area have vehicles, 37.04% of residents do not have any vehicles. This shows that most households own vehicles and the proportion of self-driving trips will be relatively high. The travel time distribution of residents in the surrounding districts in Beijing metropolitan area is calculated, as shown in Table 4.

**Fig 6:**
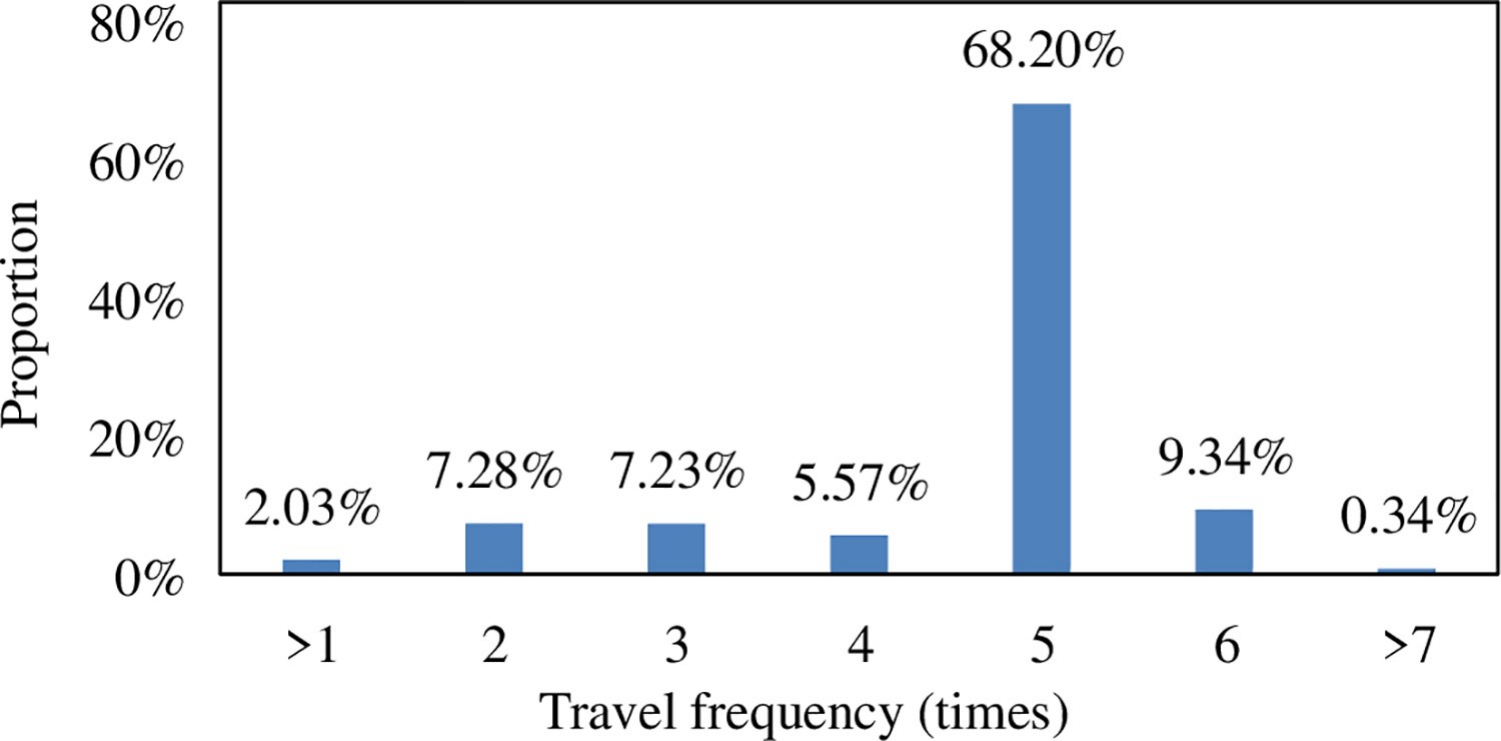
Population travel frequency distribution.in the beijing metropolitan.

According to the distribution of the travel time proportion of the surveyed persons, it is found that the travel time of all the surveyed persons is about 2 hours. The period with the highest proportion is within the range of 90–120 minutes, which is 32.86%. In the period of 120–180min, the ratio is 27.95%, and the proportion of residents in the period of 60-180min has exceeded 60%. At the same time, 8.85% of residents have spent more than 3 hours in Beijing. This shows that residents travel time is relatively long and travel time cost is high. The weekly travel frequency of residents in Beijing is calculated, and the travel frequency distribution of residents in the surrounding districts and counties in the Beijing metropolitan area, as shown in Fig 6.

**Fig 7:**
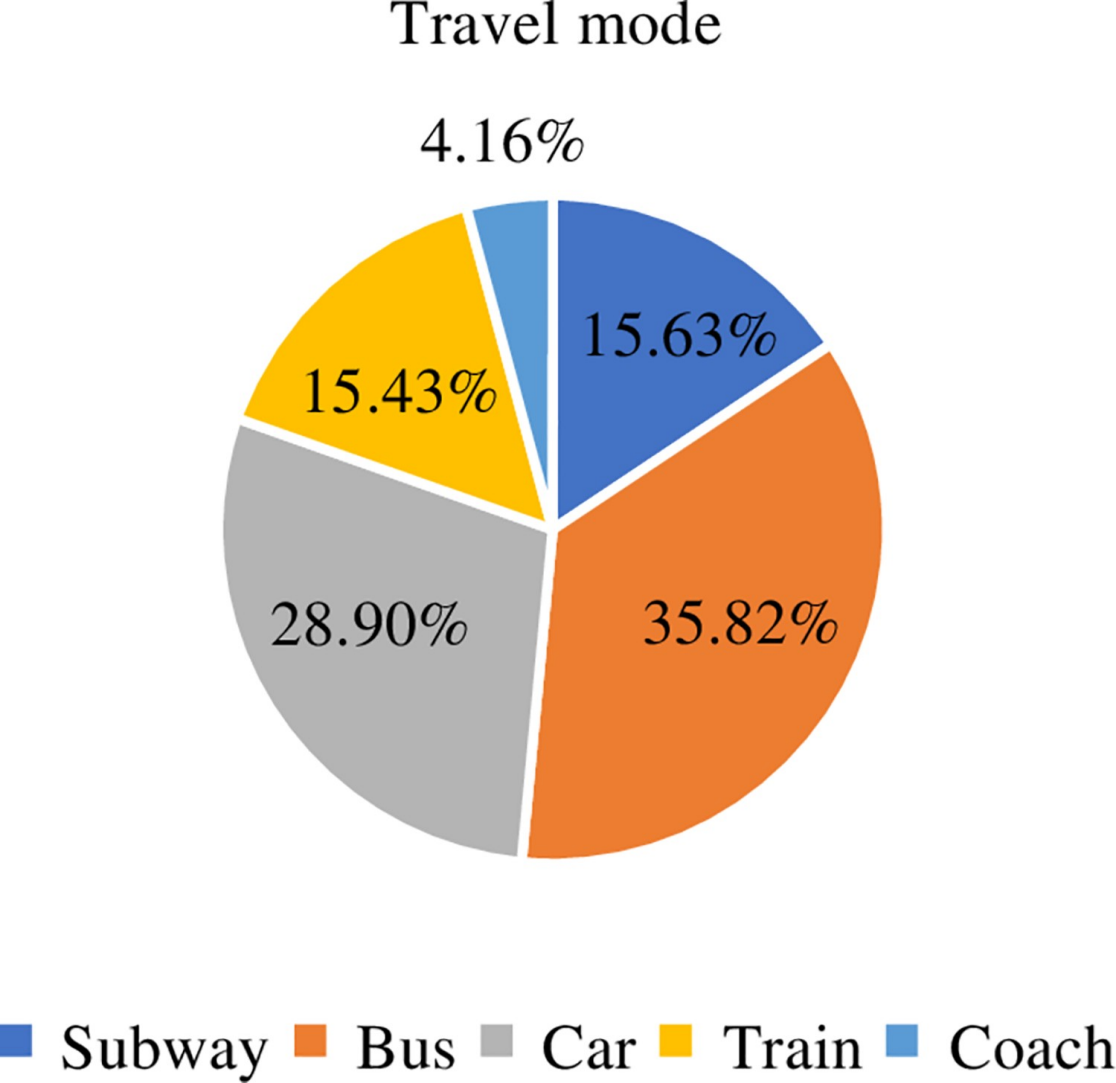
Distribution of travel modes in Beijing metropolitan area.

According to the distribution of the travel frequency ratio of the surveyed persons, it is found that the travel frequency of all the surveyed persons is more in and out of Beijing 5 times a week, with a ratio of 68.20%. It is indicated that the surveyed residents commute to Beijing more frequently. The distribution characteristics of travel modes in the Beijing metropolitan area are shown in Fig 7.

It can be seen from Fig 7, the majority of residents in the Beijing metropolitan area choose public transport, which is 35.82%. Secondly, 28.90% of residents choose cars to travel, which may be related to the long commuting distance and insufficient public transportation supply. The proportion of residents who chose the subway was 15.63%, and 15.43% of the residents chose trains. This shows that the construction of railways within the commuter circle has brought convenience for residents to commute to Beijing. It is necessary to further explore the transportation convenience brought by high-speed railways. 4.15% of residents choose the mode of coach. This group of residents are mainly located in Wuqing County with longer travel time. Based on the analysis of the travel mode and travel time in the Beijing metropolitan area, the main mode of travel for residents is public transportation, followed by cars. The commuting time is too long and travel is not convenient. Through the above analysis, it is found that the research on the travel of residents in the Beijing metropolitan area is worth exploring. The Research on the mechanism of residents’ travel characteristics and predicting travel modes can promote the sustainable development of green transportation in the Beijing metropolitan area.

## 4. Methods

It can be seen from the above analysis that residents in the Beijing metropolitan area have certain differences in the choice of travel modes, which are mainly reflected in the age of residents, travel frequency, family vehicles, family income, and travel characteristics. Based on the Softmax regression machine learning model (SR), The authors used the above factors as independent variables and travel mode as the dependent variable to build a travel mode prediction model in Beijing metropolitan area. SR has the advantages of structure simple, high classification accurate rate. SR have not been applied in the model choice field yet. It is meaningful to introduce SRBM to estimate the model performance in the mode choice field. In addition, in the field of machine learning, Support vector machine (SVM) has also shown good prediction results. The prediction model for traffic mode choice in the Beijing metropolitan area based on SVM is also constructed to compare the predicted efficiency. Due to the MNL model is mainly used in routine research to study the probability of residents’ method selection. This paper introduces MNL as a comparative model for the evaluation of prediction accuracy.

### 4.1. Variable definitions

The output of the model is the mode of transportation chosen by residents. The input is the factor that influences the choice of mode. The factors that influence the mode choice of residents is related to the individual characteristics (e.g. age, family income, and travel frequency), the family characteristics (e.g. car ownerships), the travel characteristics (e.g. travel frequency, family vehicles, travel time, travel cost, and transfer times). Where, The transfer times refers to the transfer number of times by taking the travel mode. Therefore, according to the collected data, the influencing factors considered in this paper are age, family income, travel frequency, family vehicles, travel time, travel cost and transfer times. The influencing factors are defined as variables, as shown in Table 5.

**Table 5:** Variable definition.

Factor	Variable Definition
Age (year old)	1:<18; 2:18–24; 3:25–34; 4:35–44; 5:45–49; 6:50–59; 6:>60
travel frequency	1:<1; 2:2; 3:3; 4:4; 5:5; 6:6; 7:>7
family income(RMB)	1:<1000; 2:1000–3000; 3:3000–5000; 4:5000–10000; 5:>10000
family vehicles	1: own vehicle; 2: No vehicle
travel cost (RMB)	1:<5; 2:5–10; 3:10–20; 4:20–30; 5:30–50; 6:>50
travel time (min)	1:l<30; 2:30–45; 3:45–60; 4:60–90; 5:90–120; 6:120–180; 7:>180
transfer times	1:0; 2:1; 3:2; 4:3; 5:4

### 4.2. Prediction model of travel mode based on Softmax

**Fig 8:**
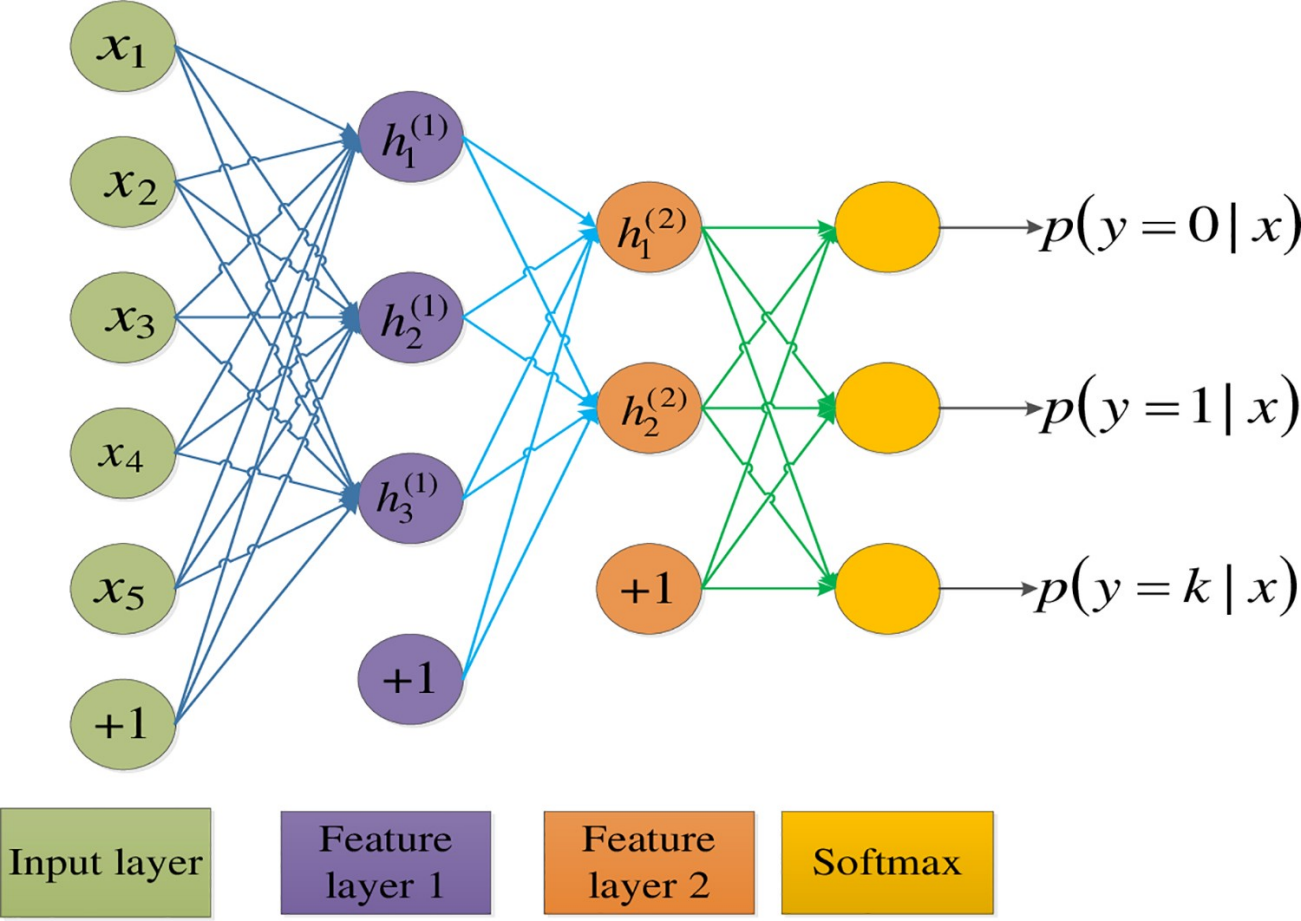
The structure of the Softmax regression machine learning model.

Softmax regression machine learning model is an extension of Logistics Regression (LR). It is different from logistic regression classification model where only two category labels can be taken. SR provides more possibilities for category labels and is suitable for multi-classification problems. The structure of SR is shown in Fig 8. The structure includes an input layer, two feature extraction layers and a classification output layer. The input feature passes through the two feature extraction layers to obtain the feature vector of the graph. It is passed into the SR classifier and only calculated by matrix multiplication. The probability of choosing each mode is output. The travel mode with the highest probability is the mode that residents ultimately choose. The output features of mode prediction in this paper are subway, bus, car, train, and coach, respectively. It is noteworthy that, since each factor has a different dimension, a normalization processing is necessary before they are input into the Softmax model.

Softmax maps the input vector from the N-dimensional space to the traffic mode. The result is given in the form of probability. The formula is shown as Eq 1

$$p_{j}=\frac{e^{\theta_{j}^{T}X}}{\sum_{k=1}^{K}e^{\theta_{j}^{T}X}}(j=1,2,\ldots ,K)$$ (1)

Where, *θ*_*k*_ = [*θ*_*k*1_,*θ*_*k*2_,⋯,*θ*_*kK*_]^*T*^ is the weight. It is the classifier parameter corresponding to the traffic mode. Total model parameters *θ* is shown as Eq 2

$$\theta =[\theta_{1}^{T},\theta_{2}^{T},\cdots ,\theta_{K}^{T}]$$ (2)

Where, *θ* is obtained by the Softmax classifier training. *θ* can be used as a parameter to calculate the probability of all possible traffic modes of the item to be classified, and then determine the selected transportation mode. A data set including *N* training samples is given: {(*x*^(1)^,*y*^(1)^), (*x*^(2)^,*y*^(2)^),…,(*x*^(*n*)^,*y*^(*n*)^)}. Where, *x* represents the input vector, and *y* is each mode of transportation. For a given test sample *x*^(*i*)^, the Softmax classifier is used to estimate the probability of belonging to each mode of transportation. The function formula is shown as Eq 3.


$$h_{\theta }(x^{\left(i\right)})=\left[\begin{array}{c} p(y^{\left(i\right)}=1)|x^{\left(i\right)};\theta \\ p(y^{\left(i\right)}=2)|x^{\left(i\right)};\theta \\ \cdot \cdot \cdot \\ p(y^{\left(i\right)}=K)|x^{\left(i\right)};\theta \end{array}\right]=\frac{1}{\sum_{k=1}^{K}e^{\theta_{k}^{T}x^{\left(i\right)}}}\left[\begin{array}{c} e^{\theta_{1}^{T}x^{\left(i\right)}}\\ e^{\theta_{2}^{T}x^{\left(i\right)}}\\ \cdot \cdot \cdot \\ e^{\theta_{K}^{T}x^{\left(i\right)}} \end{array}\right]$$ (3)

In Eq 3, *h*_*θ*_(*x*^(*i*)^) is a vector. *p*(*y*^(*i*)^ = *K*|*x*^(*i*)^;*θ*) respect the probability that *x*^(*i*)^ belongs to traffic mode *k*. The sum of *h*_*θ*_(*x*^(*i*)^) in the vector is equal to 1. For *x*^(*i*)^, The *k* corresponding to the maximum probability value is selected as the prediction result of the current travel mode of residents. The value of parameter *θ* can be obtained by minimizing the cost function of Softmax regression. First, the samples are assumed to be independent of each other. The likelihood function is shown in Eq 4.


$$L(\theta )=\prod_{i=1}^{n}\prod_{j=1}^{k}{\left(\frac{e^{\theta_{j}^{T}x^{\left(i\right)}}}{\sum_{j=1}^{K}e^{\theta_{j}^{T}x^{\left(i\right)}}}\right)}^{I(y^{\left(i\right)}=j)}$$ (4)

Where, *I*{⋅} is an indicative function. When the value is true, it is equal to 1. When the value is false, it is equal to 0. The loss function of Softmax regression is defined as Eq 5

$$J(\theta )=-\frac{1}{n}[\sum_{i=1}^{n}\sum_{j=1}^{K}I\{y^{\left(i\right)}=j\}\ln\frac{e^{\theta_{j}^{T}x^{\left(i\right)}}}{\sum_{k=1}^{K}e^{\theta_{k}^{T}x^{\left(i\right)}}}]$$ (5)

The classifier parameter *θ* can be obtained by minimizing *J*(*θ*).

### 4.3. Comparison model

**Fig 9:**
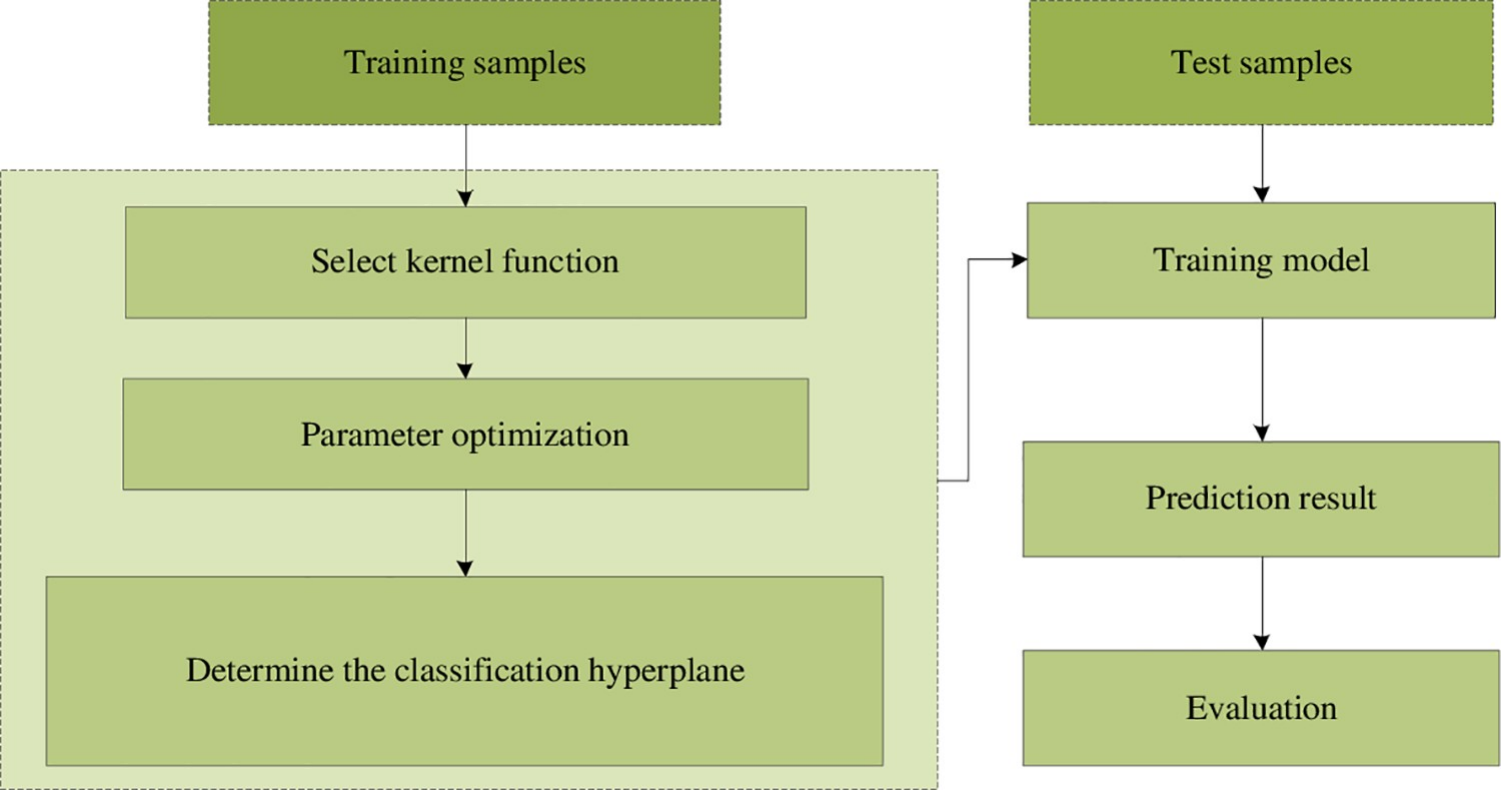
The calculated process of the SVM model.

Through the above analysis, the travel mode of residents in the Beijing metropolitan area will be affected by individuals, families, and travel characteristics. A large amount of sample data needs to be used to learn the mechanism of the choice of different characteristic attributes. SVM machine learning method has a good classification function. It can accurately classify the surveyed sample data by mapping it to a high-dimensional space, classify samples with similar characteristics into one category, and obtain travel mode predictions. Therefore it is introduced to compare the prediction efficiency with Softmax machine learning method. The calculated process of the SVM model is shown in Fig 9.

This paper uses the RBF radial basis function as the kernel function. The kernel function has the advantages of concise training, simple structure, and fast convergence speed. The kernel function can be used to solve the optimal parameters of the kernel function *r*. The kernel function formula is shown in Eq 6.


$$k(x_{i},x)=\exp(-r\frac{{\left\|x_{i}-x\right\|}^{2}}{\sigma^{2}})$$ (6)

Among them, *σ* is the variance, and *x*_*i*_ represents the *i*-th sample variable.

The Lagrangian function is introduced to construct the objective function and predict the mode of travel. The optimization function formula is shown in Eq 7.


$$\begin{array}{c} \max\sum_{i=1}^{n}\alpha_{i}-\frac{1}{2}\sum_{i,j=1}^{n}\alpha_{i}\alpha_{j}y_{i}y_{j}x_{i}^{T}x_{i}\\ s.t.\{\begin{array}{l} 0\leq \alpha \alpha_{i}\geq C,i=1,\cdots ,n\\ \sum_{i=1}^{n}\alpha_{i}y_{i}=0 \end{array} \end{array}$$ (7)

Among them, *y* represents the travel mode variable, *i* and *j* represent the sample number, *α* is the Lagrangian multiplier, *C* is the penalty factor.

In the process of training the model, it is necessary to verify and adjust the classification performance of the model. K-Cross Validation (K-CV) is adopted, which has the advantages of avoiding under-learning and over-fitting. The parameter optimization method uses a grid search method to traverse the parameters in the divided range to obtain values, and select a group of parameters with the highest accuracy rate as the optimal parameters.

Another compared model is MNL travel mode prediction model. The established model by MNL can reference Chen et al. (2015) [30].

## 5. Results

This paper uses Python programming to implement the model training and parameter evaluation of the Softmax regression traffic mode prediction model of Beijing Metropolitan area (SRBM). 3/4 of the data set is selected as the training set, and the remaining data is the validation set. The 5-fold cross-validation method was chosen to find the optimal parameters. At the same time, the above method is used for model training for SVM. In the grid search method, the value range of the penalty factor C is set to [0.01, 1000], and the range of the kernel function parameter r is set to [0.01, 1000]. After training and verification, when C = 0.01 and r = 10 are finally selected, the prediction accuracy of the validation set of the SVM model is the highest. In addition, the MNL model is calibrated with the help of STATA software, and the calibrated model is used to predict the travel mode of the original data. The prediction accuracy of the SRBM model is shown in Table 6.

**Table 6:** SRBM model prediction accuracy of 10 districts.

District	Subway	Bus	Car	Train	Coach	Total accuracy
Sanhe	89.81%	90.00%	96.38%	—	—	92.06%
Yanjiao	83.33%	95.04%	94.22%	—	—	90.86%
Langfang	—	79.38%	93.00%	93.75%	—	88.71%
Xianghe	92.25%	83.84%	94.50%	—	—	90.20%
Dachang	90.44%	95.90%	89.42%	—	—	91.92%
Gu’an	82.50%	76.27%	94.13%	—	—	84.30%
Yongqing	—	96.99%	94.72%	—	—	95.86%
Zhuozhou	—	95.44%	89.22%	15.00%	—	66.55%
Wuqing	—	—	78.17%	93.73%	94.80%	88.90%
Huailai	—	—	92.53%	95.73%	—	94.13%
Total accuracy	87.67%	89.11%	91.63%	74.55%	94.80%	—

In terms of overall accuracy, Yongqin has the highest overall accuracy of 95.86%, and the lowest prediction accuracy is in the Zhuozhou, with an accuracy rate of 66.55%. The accuracy of the prediction for mode train from Zhouzhou is only 15%. The reason is that the number of sampled data that chosen train mode is relatively small. The sampled data are not sufficient to the proposed model to explore the characteristics of the train mode. So errors occurred in mode choice predictions. From the perspective of transportation, subways, bus and coach have the highest accuracy. Among bus transportation, Yanjiao and Yongqing have the highest prediction accuracy rates of 95.9% and 96.99% respectively. Among subway transportation, Xianghe has the highest prediction accuracy of 92.25%. This shows that the establishment of the SRBM model is reasonable, it can reasonably analyze the mechanism that affects the mode choice, and can better predict the choice of transportation modes for residents.

To verify the prediction effect of the SRBM model, the SRBM prediction accuracy rate was compared with the SVM and MNL models. In this study, two indicators are selected as the evaluation function, namely, the classification prediction accuracy rate and the overall prediction accuracy rate.

### 5.1. Individual percentage of correct predictions

Individual Percentage of Correct Predictions (IPCP) refers to the proportion of accurate samples of each traffic mode to the total sample size of the traffic mode. IPCP is calculated as Eq 8.


$$IPCP=\frac{\mathrm{The}\;\mathrm{number}\;\mathrm{of}\;\mathrm{samples}\;\mathrm{with}\;\mathrm{accurate}\;\mathrm{prediction}\;\mathrm{of}\;\mathrm{the}\;\mathrm{traffic}\;\mathrm{mode}\;i}{\mathrm{The}\;\mathrm{total}\;\mathrm{sample}\;\mathrm{number}\;\mathrm{of}\;\mathrm{the}\;\mathrm{traffic}\;\mathrm{mode}\;i}$$ (8)

**Fig 10:**
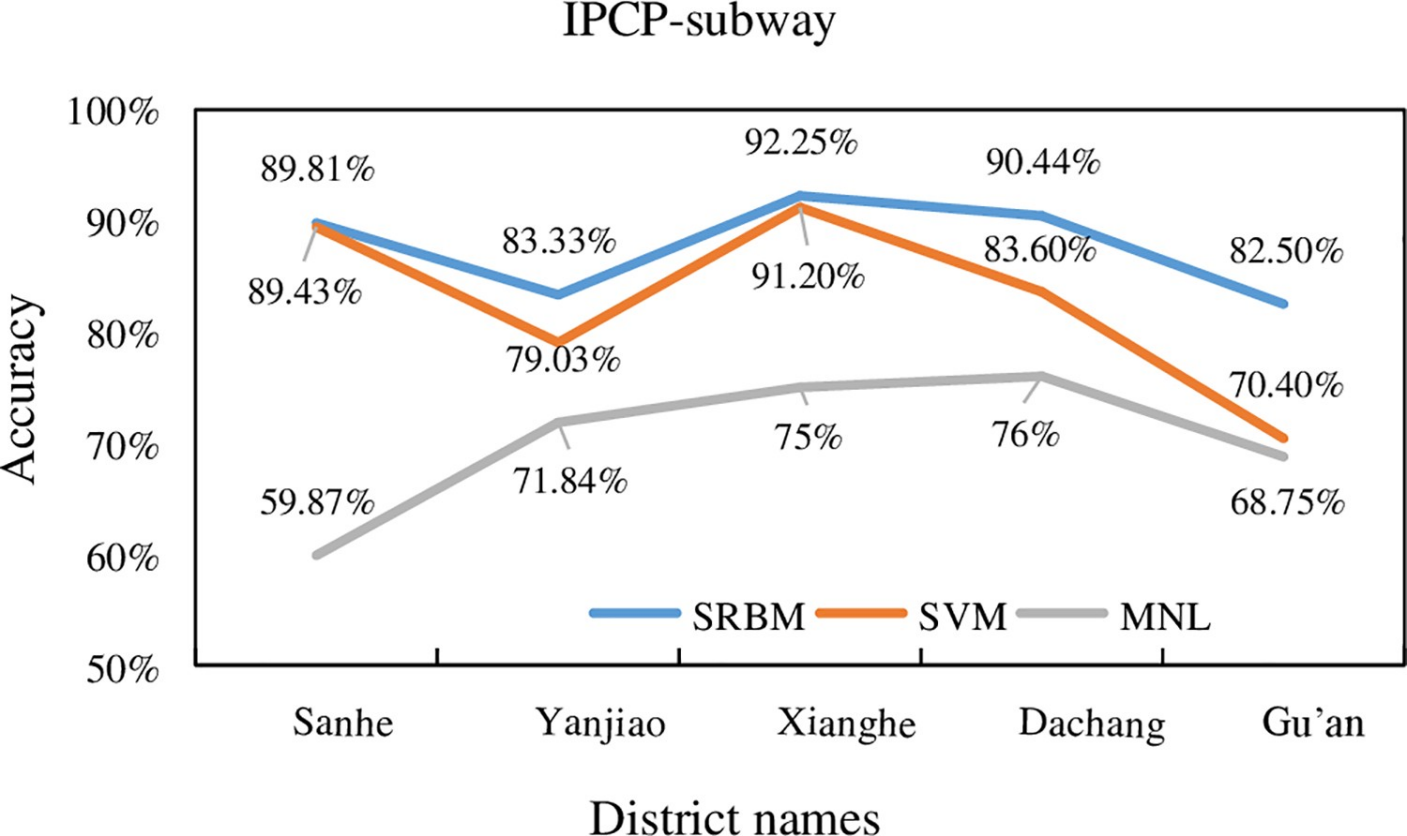
Subway accuracy of SRBM, SVM and MNL model.

**Fig 11:**
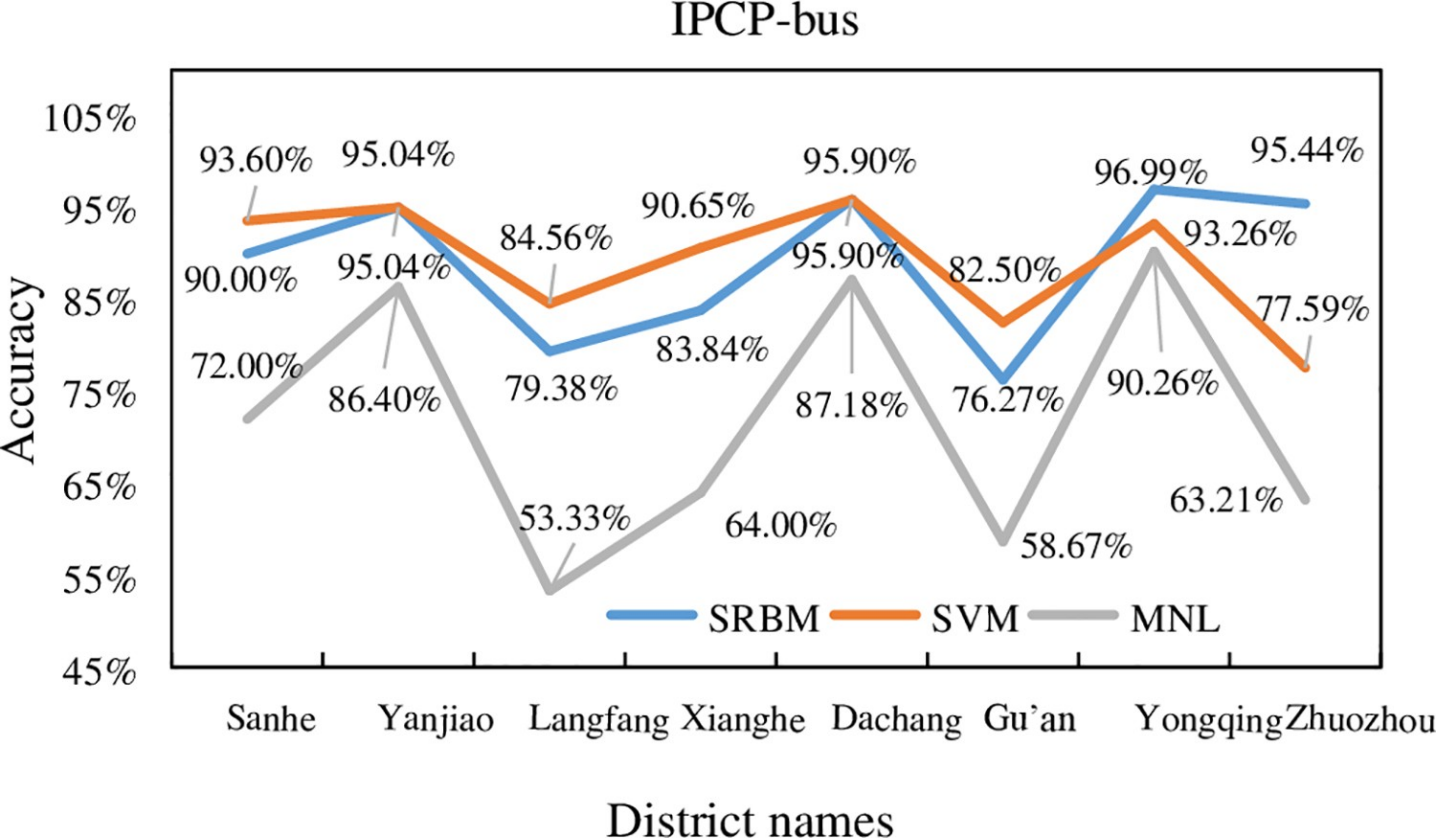
Bus accuracy of SRBM, SVM and MNL model.

**Fig 12:**
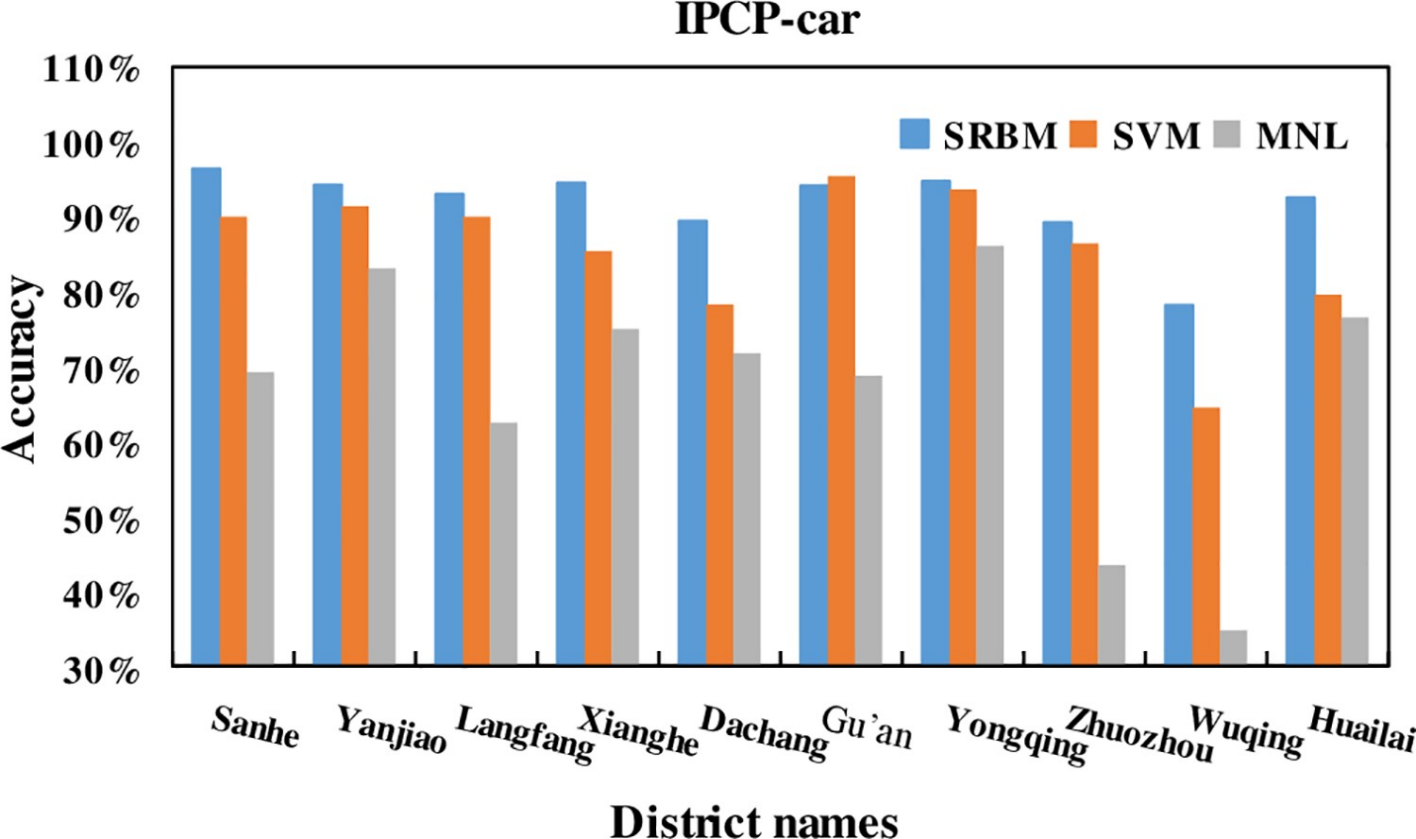
Car accuracy of SRBM, SVM and MNL model.

In this study, the three transportation modes of bus, subway and car were selected to compare the classification accuracy. The summary comparison for subway, bus, and car is shown in Figs 10–12, respectively.

The summary comparison for subway, bus, and car is shown in Table 7.

**Table 7:** Subway, bus, and car accuracy of SRBM, SVM and MNL model.

**IPCP-Subway**			
**District**	**SRBM**	**SVM**	MNL
Sanhe	89.81%	89.43%	59.87%
Yanjiao	83.33%	79.03%	71.84%
Xianghe	92.25%	91.20%	75%
Dachang	90.44%	83.60%	76%
Gu’an	82.50%	70.40%	68.75%
IPCP-Bus			
**District**	**SRBM**	**SVM**	**MNL**
Sanhe	90.00%	93.60%	72.00%
Yanjiao	95.04%	95.04%	86.40%
Langfang	79.38%	84.56%	53.33%
Xianghe	83.84%	90.65%	64.00%
Dachang	95.90%	95.90%	87.18%
Gu’an	76.27%	82.50%	58.67%
Yongqing	96.99%	93.26%	90.26%
Zhuozhou	95.44%	77.59%	63.21%
IPCP-Car			
**District**	**SRBM**	**SVM**	**MNL**
Sanhe	96.38%	90.00%	69.23%
Yanjiao	94.22%	91.38%	83.08%
Langfang	93.00%	90.00%	62.50%
Xianghe	94.50%	85.38%	75.00%
Dachang	89.42%	78.27%	71.75%
Gu’an	94.13%	95.42%	68.75%
Yongqing	94.72%	93.65%	86.11%
Zhuozhou	89.22%	86.40%	43.48%
Wuqing	78.17%	64.52%	34.78%
Huailai	92.53%	79.57%	76.52%

It can be seen from Figs 10–12, and Table 7 that the average prediction accuracy of SRBM is higher than that of SVM and MNL models. It shows that SRBM is more accurate in predicting the accuracy of traffic mode.

### 5.2. Overall percentage of correct predictions

Overall Percentage of Correct Predictions (OPCP) refers to the proportion of samples with accurate predictions of all traffic modes in the overall sample. IPCP is calculated as Eq 9.


$$OPCP=\frac{\mathrm{The}\;\mathrm{number}\;\mathrm{of}\;\mathrm{samples}\;\mathrm{with}\;\mathrm{accurate}\;\mathrm{predictions}\;\mathrm{for}\;\mathrm{all}\;\mathrm{traffic}\;\mathrm{modes}}{\mathrm{Overall}\;\mathrm{sample}\;\mathrm{size}\;\mathrm{of}\;\mathrm{all}\;\mathrm{traffic}\;\mathrm{modes}}$$ (9)

**Fig 13:**
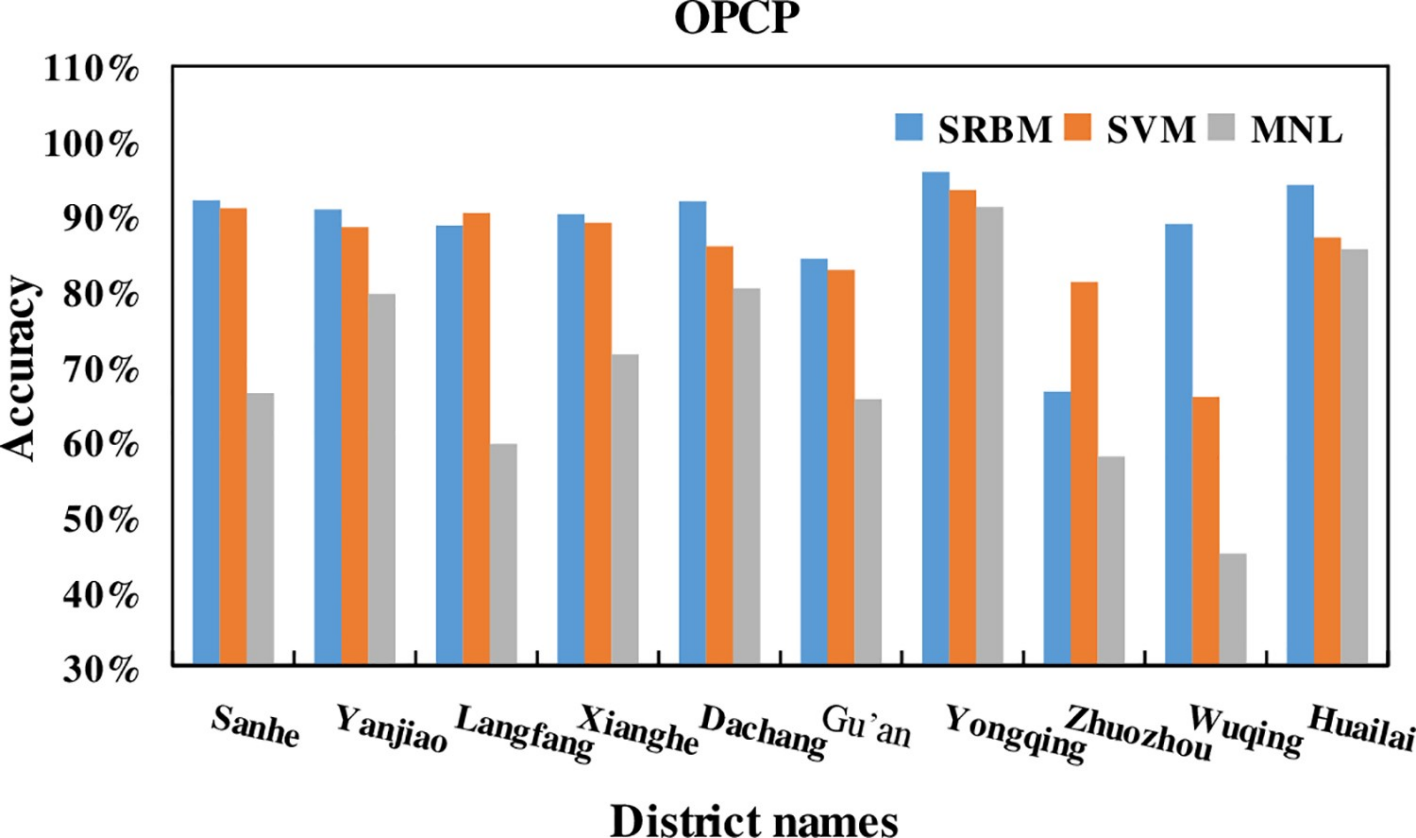
The OPCP of SRBM, SVM and MNL model.

The OPCP of SRBM, SVM and MNL model is shown in Fig 13, and Table 8.

**Table 8:** The OPCP of SRBM, SVM and MNL model.

OPCP			
District	SRBM	SVM	MNL
Sanhe	92.06%	91.01%	66.32%
Yanjiao	90.86%	88.48%	79.56%
Langfang	88.71%	90.40%	59.57%
Xianghe	90.20%	89.08%	71.49%
Dachang	91.92%	85.92%	80.33%
Gu’an	84.30%	82.77%	65.53%
Yongqing	95.86%	93.45%	91.20%
Zhuozhou	66.55%	81.16%	57.89%
Wuqing	88.90%	65.82%	44.94%
Huailai	94.13%	87.09%	85.54%

It can be seen from the Fig 13 and Table 8 that, the OPCP of SRBM in 9 out of 10 districts is higher than that of SVM and MNL models. The average OPCP of SRBM traffic mode accuracy rate in 10 districts is 88.35%. The average OPCP of traffic mode prediction accuracy rate of SVM and MNL in 10 districts is 85.52% and 70.24%, respectively. That means the average OPCP of SRBM in 10 districts is 2.83% and 18.11% higher than that of SVM and MNL, respectively. This shows that the SRBM Beijing metropolitan area residents travel mode prediction model constructed in this paper has excellent predictive ability. At the same time, it proves that the SRBM model has good applicability in the predictive analysis of the travel mode choice of residents in Beijing metropolitan area.

As can be seen that, prediction accuracy varies between different modes and different districts. The reason for this is that, there are different composition and different proportions of transport modes in different districts, meanwhile the number of survey sample is also different in different districts. Therefore, the input sample size of different modes is different, which will cause the model to have different fitting performances. Different fitting performance of the proposed model will perform various prediction accuracy between different modes (subway, bus, car, etc.) and different districts.

## 6. Conclusion

This article defines the scope of the Beijing metropolitan area in terms of time and space. With a radius of 70km, the scope of 10 districts around Beijing were selected as the Beijing Metropolitan Area. A questionnaire is designed to survey personal characteristics, family characteristics, and travel characteristics of residents living in the metropolitan area. Through the analysis of the questionnaire, it is found that the travel mode of residents in the Beijing metropolitan area has significant characteristics. The highest proportion of buses is chosen, followed by cars. As part of the public transportation, the subway has a lower proportion than cars. In addition, more than 50% of residents travel for more than 1.5 hours, which shows that the supply of public transportation is insufficient and residents travel for too long. It is suggested that in the future, the transportation policy should be tilted towards green transportation, and more public transportation should be developed to provide more convenience for residents to travel. Based on the Softmax Regression machine learning model, this paper constructs the Beijing Metropolitan Area Resident Travel Mode Prediction Model (SRBM). At the same time, SVM and MNL models are introduced to evaluate the prediction efficiency of SRBM. The results show that SRBM exhibits a high prediction accuracy, with an average prediction accuracy rate of 88.35%. The average prediction accuracy rates of SVM and NL models are only 85.52% and 70.24%, and the stability of the prediction results of each method is relatively poor. The prediction accuracy of SRBM is 2.83% and 18.11% higher than that of SVM and MNL models, respectively. This article provides new ideas for the prediction of travel modes in the Beijing metropolitan area. In the next step, the adaptability and prediction accuracy of models such as deep learning to travel modes need to be further studied. As travel distance is one of the significant factors in travel mode choice, For the further study, the factor of travel distance will be added for the mode choice problem.

## Supporting information

S1 Appendix (DOCX)

S1 File (DOC)
