# Supplementary material for: Resident travel mode prediction model in Beijing metropolitan area
Source: PLoS One. 2021 Nov 11;16(11):e0259793. doi: 10.1371/journal.pone.0259793 (PMC8588932; doi:10.1371/journal.pone.0259793)
Supplement: S1 Appendix — (DOCX) [file pone.0259793.s001.docx]

Appendix A

Resident travel mode choice survey in Beijing metropolitan area

1. What is your occupation?

○Company employee (skip to question 2) ○Student (skip to question 3) ○No occupation (skip to question 3) ○Retired (skip to question 3)

1. Do you work in Beijing?

○Yes (skip to question 9) ○No (skip to question 3)

1. How often do you go to Beijing?

○Times____a week ○Times ____a month ○Times____a year

1. What do you usually do when you go to Beijing?

○Business travel ○Seeking a doctor ○Shopping and leisure ○Sightseeing and traveling ○Visiting relatives and friends ○Others

1. What means of transportation do you generally use to get to Beijing?

○Subway (bus-to-subway, car-to-subway) ○Bus ○Car (including carpooling, online ride-hailing) ○Coach ○Train

1. How many people do you usually travel with when you go to Beijing?

○Unaccompanied ○1 person accompanied ○2 person accompanied ○3 person accompanied ○3 or more person accompanied

1. How long do you usually stay in Beijing? What to do during this period? (Multiple choice)

○Business ○Medical treatment ○Shopping and leisure ○Visiting relatives and friends ○Other

1. What is your main mode of transportation to work? What is the secondary mode of transportation?

○Subway (including bus-to-subway, car-to-subway) ○Bus ○Car (including carpooling, online ride-hailing) ○Coach ○Train

1. What is your average time from going out to work?

○Within 30 minutes ○30-45 minutes ○45-60 minutes ○60-90 minutes ○90-120 minutes ○120-180 minutes ○180 minutes and above

1. How often do you commute to Beijing in a week?_________
2. What is your household registration type?

○Beijing ○Hebei ○Tianjin ○Others

1. What is your age group?

○Under 18 years old ○18-24 years old ○25-34 years old ○35-44 years old ○45-49 years old ○50-59 years old ○60 years old and above

1. Is there any vehicle in the family that you can use freely?

○No ○Yes

1. What is the vehicle license plate? (Multiple choice)

○Beijing ○Hebei ○Tianjin ○Others

1. What are the expenses you can freely spend every month (excluding expenses such as mortgages and fixed expenses)?

○Below 1000 RMB ○1000-3000 RMB ○3000-5000 RMB ○5000-10000 RMB ○More than 10,000 RMB

1. If the intercity railway or other types of rail transportation from your city to Beijing will be opened in the future, will you take it?

○Yes ○N
