# Supplementary material for: Resident travel mode prediction model in Beijing metropolitan area
Source: PLoS One. 2021 Nov 11;16(11):e0259793. doi: 10.1371/journal.pone.0259793 (PMC8588932; doi:10.1371/journal.pone.0259793)
Supplement: S1 File — (DOC) [file pone.0259793.s002.doc]

Supporting information

[Table 1. Comparison of Population and Traffic Demand Characteristics Between Metropolitan Areas and Urban Agglomerations. 1](#__RefHeading___Toc2242)

[Table 2. Comparison of the Scope and Structural Characteristics of Metropolitan Areas and Urban agglomerations. 2](#__RefHeading___Toc14737)

[Table 3. Prediction of Social and Economic Development in Beijing 2](#__RefHeading___Toc15424)

[Table 4. Population Vehicle Ownership Distribution and travel time distribution in Beijing Metropolitan area. 2](#__RefHeading___Toc2387)

[Table 5. Variable Definition. 2](#__RefHeading___Toc9574)

[Table 6. SRBM Model prediction Accuracy of 10 Districts. 2](#__RefHeading___Toc32567)

[Table 7. Subway, bus, and car accuracy of SRBM,SVM and MNL Model. 3](#__RefHeading___Toc32731)

[Table 8. The OPCP of SRBM, SVM and MNL model. 3](#__RefHeading___Toc19225)

[Fig 1. Urban Agglomeration and Metropolitan Coordinating Region Structure 4](#__RefHeading___Toc23438)

[Fig 2. Beijing metropolitan area definition. 4](#__RefHeading___Toc1680)

[Fig 3. Survey Sample Size Chart - Artificial Questionnaire. 5](#__RefHeading___Toc6041)

[Fig 4. Survey Sample Size Chart - Online Questionnaire. 5](#__RefHeading___Toc30910)

[Fig 5. Beijing Metropolitan Population Age Distribution. 5](#__RefHeading___Toc31040)

[Fig 6. Beijing Metropolitan Population Disposable Income Distribution. 6](#__RefHeading___Toc10718)

[Fig 7. Beijing Metropolitan Population Travel Frequency Distribution Table. 6](#__RefHeading___Toc26567)

[Fig 8. Distribution of travel modes in Beijing metropolitan area. 6](#__RefHeading___Toc19502)

[Fig 9. The structure of the Softmax regression machine learning model. 7](#__RefHeading___Toc23618)

[Fig 11. Subway accuracy of SRBM,SVM and MNL Model. 7](#__RefHeading___Toc27932)

[Fig 12. Bus accuracy of SRBM, SVM and MNL Model. 8](#__RefHeading___Toc19587)

[Fig 13. Car accuracy of SRBM,SVM and MNL Model. 8](#__RefHeading___Toc14556)

[Fig 14. The OPCP of SRBM, SVM and MNL model. 8](#__RefHeading___Toc21202)

**Table 1. Comparison of Population and Traffic Demand Characteristics Between Metropolitan Areas and Urban Agglomerations.**

| **Main characteristics** | **Population size** | **Transportation demand characteristics** |
| --- | --- | --- |
| Metropolitan area | total population ≥ 5 million  Central city population  ≥ 1 million | commuting transportation needs |
| Urban agglomeration | total population ≥ 25 million | the transportation of raw materials and semi-finished products between the upstream and downstream industrial chains;  A large number of business trips between cities |

**Table 2. Comparison of the Scope and Structural Characteristics of Metropolitan Areas and Urban agglomerations.**

| **Main characteristics** | **Scope scale** | **spatial structure characteristics** |
| --- | --- | --- |
| Metropolitan area | Combined commuting time and commuting expenses, Generally around 70km | layered structure |
| Urban agglomeration | Huge space | circle layer + corridor structure |

**Table 3. Prediction of Social and Economic Development in Beijing**

| **Index** | **2015** | **2016** | **2017** | **2018** | **2019** | **2020** |
| --- | --- | --- | --- | --- | --- | --- |
| Gross product (billions of RMB) | 2296.9 | 2457.9 | 2638.4 | 2820.8 | 2998.7 | 3177.9 |
| GDP growth rate in the actual area | 6.9% | 6.5% | 6.3% | 5.6% | 5.4% | 4.7% |
| Personal consumption (billions of RMB) | 849.5 | 934.8 | 1024.9 | 1124.2 | 1227.2 | 1333.7 |
| Population (million) | 22.8 | 22.4 | 22.8 | 23.1 | 23.4 | 23.6 |
| Gross the per capita area (RMB) | 100639 | 109693 | 115956 | 122273 | 128377 | 134539 |
| Actual pay (year-on-year) | 7.5% | 7.1% | 6.3% | 5% | 4.5% | 4.5% |

**Table 4. Population Vehicle Ownership Distribution and travel time distribution in Beijing Metropolitan area.**

| Beijing metropolitan passenger car ownership distribution | | | | | | | | | |
| --- | --- | --- | --- | --- | --- | --- | --- | --- | --- |
| **Vehicle presence** | | | **No vehicle** | | | | **Own vehicle** | | |
| Proportion | | | 37.04% | | | | 62.96% | | |
| Beijing metropolitan resident travel time distribution | | | | | | | | | |
| **Time period** | **<30 min** | **30-45 min** | | **45-60 min** | **60-90 min** | **90-120 min** | | **120-180min** | **>180min** |
| Proportion | 1.17% | 1.03% | | 13.79% | 14.35% | 32.86% | | 27.95% | 8.85% |

**Table 5. Variable Definition.**

| **Factor** | **Variable Definition** |
| --- | --- |
| Age (year old) | 1:<18; 2:18-24; 3:25-34; 4:35-44; 5:45-49; 6:50-59; 6:>60 |
| travel frequency | 1:<1; 2:2; 3:3; 4:4; 5:5; 6:6; 7:>7 |
| family income(RMB) | 1:<1000; 2:1000-3000; 3:3000-5000; 4:5000-10000; 5:>10000 |
| family vehicles | 1: own vehicle; 2: No vehicle |
| travel cost (RMB) | 1:<5; 2:5-10; 3:10-20; 4:20-30; 5:30-50; 6:>50 |
| travel time (min) | 1:l<30; 2:30-45; 3:45-60; 4:60-90; 5:90-120; 6:120-180; 7:>180 |
| transfer times | 1:0; 2:1; 3:2; 4:3; 5:4 |

**Table 6. SRBM Model prediction Accuracy of 10 Districts.**

| **District** | **Subway** | **Bus** | **Car** | **Train** | **Coach** | **Total accuracy** |
| --- | --- | --- | --- | --- | --- | --- |
| Sanhe | 89.81% | 90.00% | 96.38% | — | — | 92.06% |
| Yanjiao | 83.33% | 95.04% | 94.22% | — | — | 90.86% |
| Langfang | — | 79.38% | 93.00% | 93.75% | — | 88.71% |
| Xianghe | 92.25% | 83.84% | 94.50% | — | — | 90.20% |
| Dachang | 90.44% | 95.90% | 89.42% | — | — | 91.92% |
| Gu’an | 82.50% | 76.27% | 94.13% | — | — | 84.30% |
| Yongqing | — | 96.99% | 94.72% | — | — | 95.86% |
| Zhuozhou | — | 95.44% | 89.22% | 15.00% | — | 66.55% |
| Wuqing | — | — | 78.17% | 93.73% | 94.80% | 88.90% |
| Huailai | — | — | 92.53% | 95.73% | — | 94.13% |
| Total accuracy | 87.67% | 89.11% | 91.63% | 74.55% | 94.80% | — |

**Table 7. Subway, bus, and car accuracy of SRBM,SVM and MNL Model.**

| **IPCP-Subway** | | | |
| --- | --- | --- | --- |
| **District** | **SRBM** | **SVM** | MNL |
| Sanhe | 89.81% | 89.43% | 59.87% |
| Yanjiao | 83.33% | 79.03% | 71.84% |
| Xianghe | 92.25% | 91.20% | 75% |
| Dachang | 90.44% | 83.60% | 76% |
| Gu’an | 82.50% | 70.40% | 68.75% |
| IPCP-Bus | | | |
| **District** | **SRBM** | **SVM** | **MNL** |
| Sanhe | 90.00% | 93.60% | 72.00% |
| Yanjiao | 95.04% | 95.04% | 86.40% |
| Langfang | 79.38% | 84.56% | 53.33% |
| Xianghe | 83.84% | 90.65% | 64.00% |
| Dachang | 95.90% | 95.90% | 87.18% |
| Gu’an | 76.27% | 82.50% | 58.67% |
| Yongqing | 96.99% | 93.26% | 90.26% |
| Zhuozhou | 95.44% | 77.59% | 63.21% |
| IPCP-Car | | | |
| **District** | **SRBM** | **SVM** | **MNL** |
| Sanhe | 96.38% | 90.00% | 69.23% |
| Yanjiao | 94.22% | 91.38% | 83.08% |
| Langfang | 93.00% | 90.00% | 62.50% |
| Xianghe | 94.50% | 85.38% | 75.00% |
| Dachang | 89.42% | 78.27% | 71.75% |
| Gu’an | 94.13% | 95.42% | 68.75% |
| Yongqing | 94.72% | 93.65% | 86.11% |
| Zhuozhou | 89.22% | 86.40% | 43.48% |
| Wuqing | 78.17% | 64.52% | 34.78% |
| Huailai | 92.53% | 79.57% | 76.52% |

**Table 8. The OPCP of SRBM, SVM and MNL model.**

| **OPCP** | | | |
| --- | --- | --- | --- |
| **District** | **SRBM** | **SVM** | MNL |
| Sanhe | 92.06% | 91.01% | 66.32% |
| Yanjiao | 90.86% | 88.48% | 79.56% |
| Langfang | 88.71% | 90.40% | 59.57% |
| Xianghe | 90.20% | 89.08% | 71.49% |
| Dachang | 91.92% | 85.92% | 80.33% |
| Gu’an | 84.30% | 82.77% | 65.53% |
| Yongqing | 95.86% | 93.45% | 91.20% |
| Zhuozhou | 66.55% | 81.16% | 57.89% |
| Wuqing | 88.90% | 65.82% | 44.94% |
| Huailai | 94.13% | 87.09% | 85.54% |


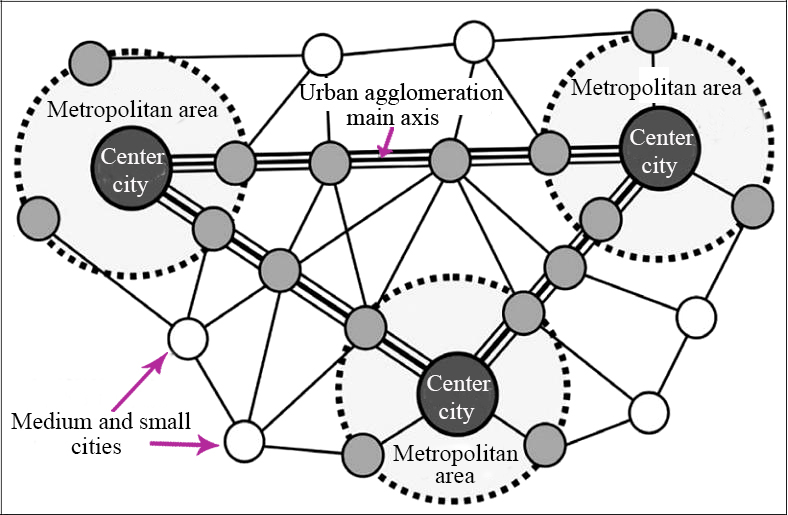


**Fig 1. Urban Agglomeration and Metropolitan Coordinating Region Structure**


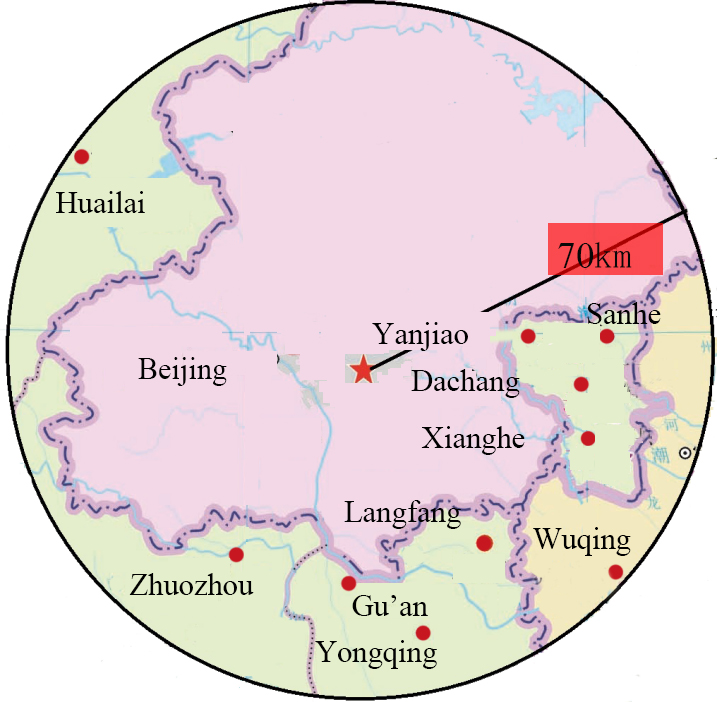


**Fig 2. Beijing metropolitan area definition.**

**Fig 3. Survey Sample Size Chart - Artificial Questionnaire.**

**Fig 4. Survey Sample Size Chart - Online Questionnaire.**

**Fig 5. Beijing Metropolitan Population Age Distribution.**

**Fig 6. Beijing Metropolitan Population Disposable Income Distribution.**

**Fig 7. Beijing Metropolitan Population Travel Frequency Distribution Table.**

**Fig 8. Distribution of travel modes in Beijing metropolitan area.**

**Fig 9. The structure of the Softmax regression machine learning model.**


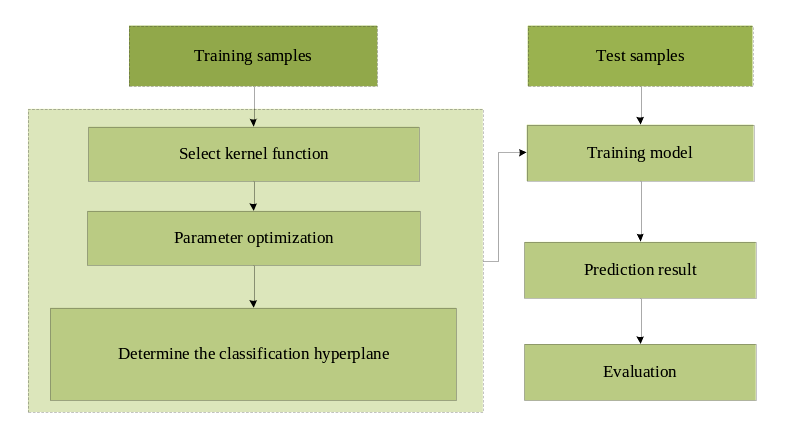


**Fig 10. The calculated process of the SVM model**

**Fig 11. Subway accuracy of SRBM,SVM and MNL Model.**

**Fig 12. Bus accuracy of SRBM, SVM and MNL Model.**

**Fig 13. Car accuracy of SRBM,SVM and MNL Model.**

**Fig 14. The OPCP of SRBM, SVM and MNL model.**
